# Fingerprints, barcode sequences and quasi-phylogenies–Tools for analysing polyphonic music

**DOI:** 10.1371/journal.pone.0280478

**Published:** 2023-03-02

**Authors:** Gerhard Rambold, Dieter Neubacher, Siegfried Schießl

**Affiliations:** 1 University of Bayreuth, Bayreuth, Germany; 2 SNSB IT Center, München, Germany; 3 ARION Publishing, Baden-Baden, Germany; Georgia Institute of Technology, UNITED STATES

## Abstract

A data flow is presented for visualising the evolution of elementary structures of polyphonic music from early Baroque to late Romantic, using quasi-phylogenies based on fingerprint diagrams and barcode sequence data of 2-tuples of consecutive vertical pitch class sets (*pcs*). The present methodological study, which sees itself as a proof of concept for a data-driven approach, uses examples of music from the Baroque, the Viennese School and the Romantic era to show that such quasi-phylogenies can be generated from multi-track MIDI (v. 1) files that largely correspond to the eras and the chronology of compositions and composers. The method presented is considered to have the potential to support the analysis of a wide range of musicological questions. In the context of collaborative work on quasi-phylogenies of polyphonic music, a public data archive could be established that provides multi-track MIDI files with contextual data.

## Introduction

Systematic branches of science such as biology, linguistics and musicology need instruments to describe and compare their research objects according to uniform criteria. In the systematic sciences, these criteria depend on the accessibility and recordability of the characteristics, which in turn depend on the methodology. In biological systematics, it was initially phenotypic, i.e. morpho-anatomical, features that were used, for example, to distinguish species. Later, characteristics at the cellular and chemical level were added. Finally, it was genotypic characteristics (DNA sequence data) that led to the further development of classifications. DNA barcoding genes, as the name suggests, are used to identify individual organisms and play an important role to characterise and compare organisms or groups of organisms [1]. These genes or gene segments occur in all representatives of the groups of organisms under consideration and thus enable comparison.

Genes or gene segments used as species barcoding genes or species barcodes are also used to reconstruct the evolution of species or taxa. For this purpose, the sequence data are arranged in a matrix and the dissimilarities of the individual sequences are visualised as phylograms using various algorithms and parameters [2]. The resulting topologies provide insight into the evolution of certain traits within a particular phylogenetic group [3] and can be used as a basis for the classification of research objects with the taxonomic levels of species, genus, family, order, etc., as in systematic biology [4]. Ultimately, entire object or class hierarchies can be built up like evolutionary classification systems.

Barcoding genes fulfil the following criteria: 1) They are intrinsic properties that cannot be changed or only insignificantly changed by environmental impacts, as is the case with the genome (persistence, consistency). 2) They are measurable properties that are intrinsic to the object and for which repeated measurements lead to the same result (repeatability, reproducibility). 3) The underlying properties occur in all objects to be compared (universality). 4) It is possible to represent the characteristic values as a sequence of code elements whose elements can be positioned, e.g. in a so-called alignment, in such a way that corresponding, i.e. homologous, elements lie in the same horizontal positions, so that a data matrix is created that can subsequently be processed for visualisation and analysis (processability, comparability).

**Fig 1:**
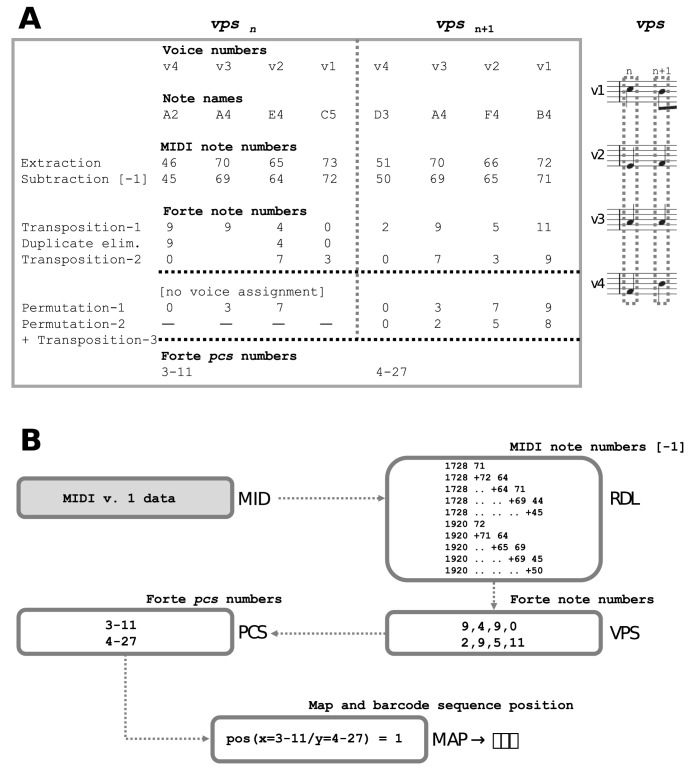
Data flow from multi-track MIDI files to RAMEAU intermediate processing files for subsequent generation of fingerprints, barcodes and the alignment as used for tree generation. **A.** Data flow from a MIDI input file to a *pcs* listing (all in UTF-8 encoding format). [MID: MIDI; the input files must follow the multi-track MIDI standard (v. 1); RDL: Reduced Data Listing files provide all basic information required for analyses based on VPS files, supplying all information concerning tone = pitch (as MIDI note numbers), duration of tone for every track = voice; VPS: Vertical Pitch Set files include successive vertical pitch sets; PCS: Pitch Class Set files include the successive vertical pitch sets (as ‘Forte name/numbers’]. **B.** Data flow schema from a multi-track MIDI input file to a MAP fingerprint file and to SEQ barcode sequence data (to be copied or exported from the cmd window under local MS Windows).

Among the systematic sciences, linguistics and musicology are predestined for a similar approach, especially since both have as their object of research the information encoded in a limited number of different letters or notes, respectively. In polyphonic (including homophonic) music (representable by 12 half tones), quite analogously both a quasi ‘phenotypic’ and a ‘genotypic’ structural level can be distinguished in the respective works. Phenotypical characteristics of music compositional works are all those that can be influenced by instrumentation and interpretation, i.e. key, rhythm (incl. tempo) as well as expression (incl. articulation and timbre). Genotypic in a certain sense, on the other hand, are elementary tonal structures that cannot be easily influenced without affecting the compositional identity, such as melody and harmony (without key), and which can be described on the basis of the set theory of music [5]. In a data flow, sets of tones or pitches coded in multi-track MIDI v. 1 format (https://www.midi.org/specifications) can be transformed into each other and assigned to pitch class sets (*pcs*) by elementary transformation procedures such as transposition, permutation and mirroring (Fig 1A and 1B). In other words, defined *pcs* are represented by pitch sets that can be transformed into each other by the above-mentioned transformation procedures. Thus there is a limited number of *pcs*, which are named or numbered according to a classification system with names or numbers by Forte (1973) [5].

**Fig 2:**
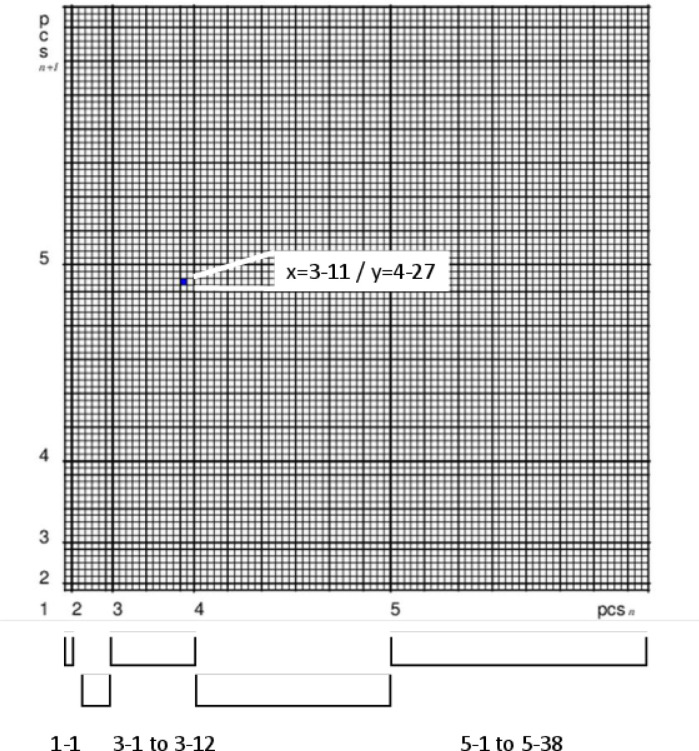
Schematic of creation of a fingerprint grid diagram of *pcs*_n_- *pcs*_n+1_ 2-tuples. [The consecutive *pcs* (corresponding to chordal links) are coded and plotted as ‘Forte name/numbers’ (i.e. *pcs* 1–1, 2–1 to 2–6, 3–1 to 3–12 etc.) of the initial *pcs*_n_ = right hand value (x) and the name/number of the target *pcs*_n+1_ = high value (y)].

Vertical pitch sets (corresponding to chords but independent of key and permutation) can be assigned to a *pcs* at any point of a pitch change in polyphonic compositional works. According to chordal links, the consecutive *pcs* (*pcs*_n_ → *pcs*_n+1_) can be represented and plotted as ordered pairs, i.e. 2-tuples in a grid diagram, where the name/number of the initial *pcs* (*pcs*_n_) is the right-hand value and the subsequent *pcs* (*pcs*_n+1_) the high value. The target *pcs* becomes the initial *pcs* in the subsequent step. The procedure for creating these diagrams is shown in Fig 2.

**Fig 3:**
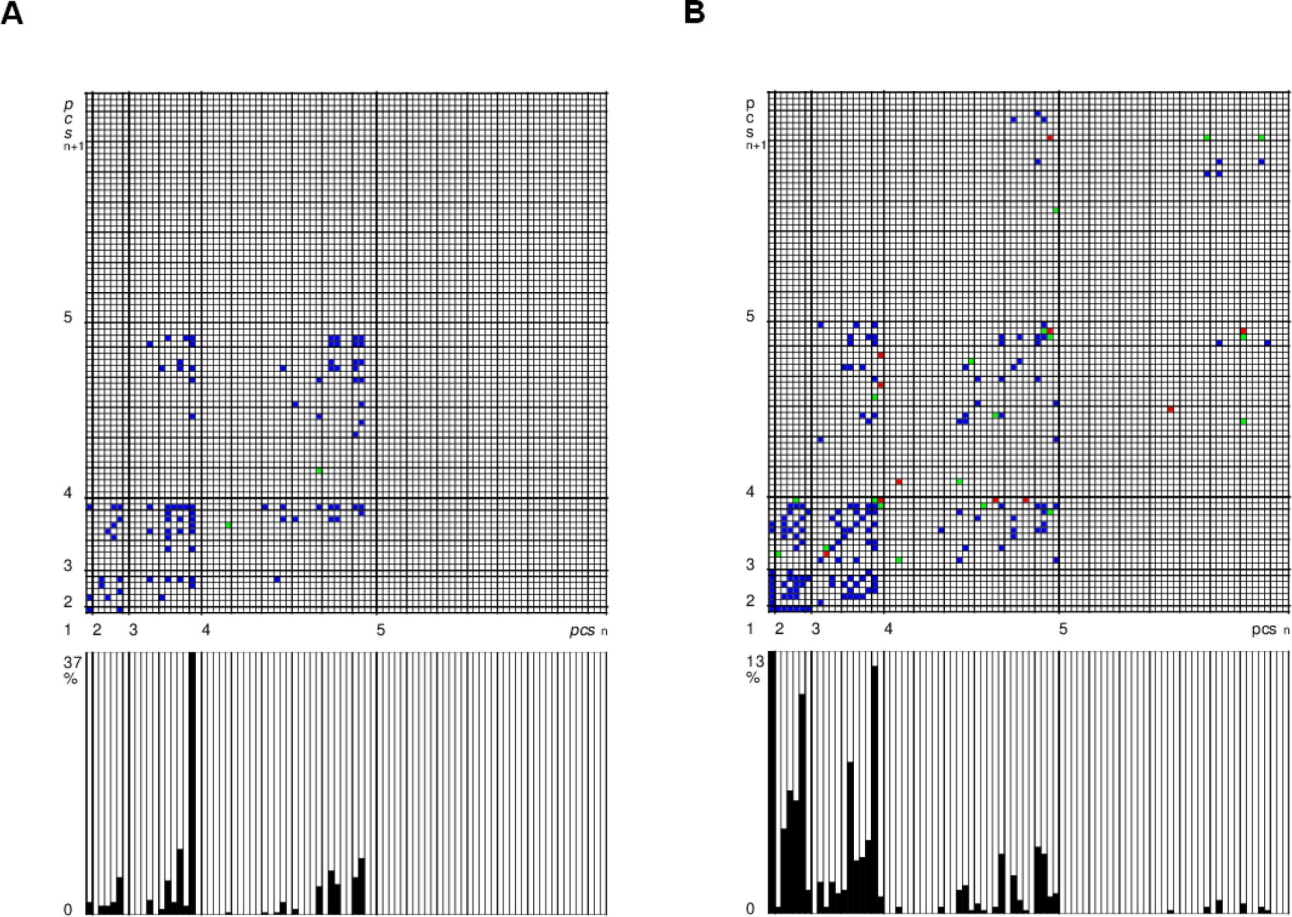
Fingerprint grid diagrams of *pcs*_n_- *pcs*_n+1_ 2-tuples and relative *pcs* abundances of examined compositional works. [**A:** D. Buxtehude 1675−76; **B:** A. Bruckner, 1878; based on vertical pitch set links (tuples of consecutive vertical *pitch class sets*) coded as pitch class sets (*pcs*). Consecutive ordered pairs of *pcs* both being extentionally diatonic (incl. intentionally chromatic) (**blue**); both *pcs* being extentionally chromatic (i.e. non-diatonic) (**red**); a *pcs* being extentionally diatonic (incl. intentionally chromatic), followed or preceded by a *pcs* being extentionally chromatic (i.e. non-diatonic) (**green**). Bar charts of relative abundances of occurring *pcs* from *pcs* 1–1 to *pcs* 5–38 at the bottom.] Symphony no. 5 Symphony no. 5.

After repeatedly applying *pcs*_n_-*pcs*_n+1_ 2-tuples according to their respective Forte names/numbers, characteristic patterns result for the respective compositional works or parts thereof. The resulting diagrams can be understood as signatures or fingerprints on the genotypic level in the sense described above. They provide information about the type and number of different fundamental pairs of *pcs* and show the occurrence of connections between diatonic pitch classes (i.e. extentionally diatonic and intentionally chromatic connections) on the one hand (cells coloured blue in Fig 3), and of extentionally chromatic pitch class connections on the other (red), as well as of the connections between both types of consecutive *pcs* 2-tuples (green). Abundances of occurring *pcs* are provided as well (Fig 3, bottom). The resulting diagrams appear to be typical for certain composition styles and composers [6,7]. Since the greater of these two studies was published in German and not in a scientific journal, the approach has received little attention, so it has not yet been taken up and developed further. Comparing the two examples from two eras given in Fig 3A and 3B and considering the total of fingerprint grid diagrams generated in this study (S2, S3A–S3J, S4A–S4AF and S5 Figs), it is clear that the patterns follow certain basic structures. A purely random selection of vertical pitch sets would result in a diagram with a uniform distribution of grid points (data not shown).

In the present study, we have further developed the approaches of previous works [5–7] to enable the construction of ‘quasi-phylogenetic’ trees (in analogy to ‘phylogenetic’ trees in evolutionary biology) for direct comparison of music compositional works based on alignments of barcode sequence data of 2-tuples of consecutive vertical pitch class sets (*pcs*). The aim of the present study was to create quasi-phylogenetic trees where a) the compositions and/or composers of at least one era form a quasi-monophyletic group and b) that within the quasi-monophyla it should be possible to read to some extent a chronology of the compositions with good statistical support values.

## Methodology

The music notation software Sibelius [8] was used to digitise the music compositional works, and MIDI files in version 1 were exported for subsequent analysis (S1 Fig). From these MIDI files, the open source analysis software and processual pipeline RAMEAU v. 1.1, released under the GNU General Public License v. 3.0, was used to create a series of intermediate files containing the listings of raw data (*.rdl), of vertical pitch sets (*.vps), of pitch class sets (*.pcs) (S6 Fig), and to create fingerprint grid diagrams (*.map.ps) of the individual music compositional works or parts thereof, respectively.

To generate quasi-phylogenetic trees from the fingerprint data, the binary values (absent-present) of the cells (= positions 1−n, i.e. ‘1 to n’) of the (two-dimensional) grid must be brought into a single (one-dimensional) line (y = 1, x = 1−n → y = 2, x = 1−n → y = 3, x = 1−n → … y = n, x = 1−n), which now represents a binary string and the ‘barcode sequence’ of the selected music compositional work (or parts thereof). A sequence of grid points may be coded with ‘1’ for the filled cells in the diagram and with ‘0’ for empty cells. A sequence of grid cell points from a grid diagram alignment based on *pcs* cardinalities 1 to 5 (*pcs* 1–1, *pcs* 2–1 to *pcs* 2–6, *pcs* 3–1 to *pcs* 3–12, *pcs* 4–1 to *pcs* 4–29, and *pcs* 5–1 to *pcs* 5–38) therefore has a length of 86 × 86 = 7,396 positions or digits. Alignments based on *pcs* cardinalities 1 to 6 (i.e. including *pcs* 1–1 to *pcs* 6–50) even comprise (86 + 50) × (86 + 50) = 18,496 positions as in the present study (S7A Fig). However, the corresponding grid diagrams of the given examples only consider cardinalities up to 5. As in biological phylogenetics, quasi-phylogenetic trees can now be calculated from such alignments using various methods.

As use case, a total of 28 (20) compositions by 14 (10) composers from the Baroque era, 10 compositions by 4 composers from the Viennese School, and 32 compositions by 16 composers from the Romantic era were selected for creating grid diagrams (numbers of works considered in the quasi-phylogenies in brackets). The names of the compositions and composers as well as the years of composition are listed in Table 1A–1C. For reasons of comparability and practicability, the first 20 bars (without the upbeat) mostly of the respective first movement of these compositions were included. The resulting fingerprints are provided as S2, S3A−S3J, S4A–S4AF and S5 Figs.

**Table 1:** A, B, C. Examples of music compositional works from the Baroque era, the Viennese School and the Romantic era used for fingerprinting and the generation of quasi-phylogenies. [First 20 bars (without upbeat) of the first movement considered].

**A) Baroque era**			
	**Composer**	**Composition**	**Year of composition**
A	Carl Philipp Emanuel Bach	Symphony, G major, H 648, Wq 173, 1^st^ movement	1741
**B**		Flute Concerto, D minor, H 484, Wq 22, 1^st^ movement	1747
**C**	Johann Sebastian Bach	Komm, Heiliger Geist, Herre Gott, G major, BWV 226/2 ChS	1729
**D**		Die Kunst der Fuge, Contrapunctus alla Decima, A minor, BWV 1080, no. 10	1748−50
**E**	Dieterich Buxtehude	Nun bitten wir den heiligen Geist, G major, BuxWV 209	1675−76
**F**		Trio Sonata, E major, BuxWv 264	1696
**G**	Marc-Antoine Charpentier	Ave Regina, C major, H 19	1672
**H**		Confitebor tibi Domine, A minor, H 200	1680
**I**	Arcangelo Corelli	Concerto Grosso, G minor, op. 6, no. 8, 1^st^ movement	1690
**J**		Trio Sonata, G minor, op. 4, no. 2	1694
**K**	François Couperin	Tantum ergo, G minor	[midlife ~1700]
**L**		Lauda Sion Salvatorem, G minor	[midlife ~1700]
**M**	Georg Friedrich Händel	Concerto Grosso, D minor, op. 3, no. 5, HWV 316, 1^st^ movement	1715−18
**N**		Organ concert, G minor, op. 7 no. 5, HWV 310	1750
**O**	Jean-Baptiste Lully	Ballet de la Raillerie, G major, LWV 11	1659
**P**		Amadis de Gaule, Overture, G major	1684
**Q**	Claudio Monteverdi (Renaissance era)	Madrigal, Qu’io non t’ami, cor mio, C major, SV 70	1592
**R**		Deus tuorum militum, C major	1641
**S**	Johann Pachelbel	Canon, D major	1680
**T**		Canon, Choral-Prelude, Durch Adams Fall ist ganz verderbt, D major, P 103	1694
**U**	Domenico Scarlatti	Keyboard Sonata, D minor, K 191, 1^st^ movement	[midlife ~1721]
**V**		Keyboard Sonata, F minor, K 183, 1^st^ movement	[midlife ~1721]
**W**	Heinrich Schütz	Jubilate Deo omnis terra, G major, SWV 262	1629
**X**		Das Wort ward Fleisch, C major, SWV 385	1648
**Y**	Georg Philipp Telemann	Concerto primo, G major, TWV 43-G1	1730
**Z**		Concerto à 4, D minor, TWV 43, D4, 2^nd^ movement	, 1752
**AA**	Antonio Vivaldi	Concerto, C major, RV 554	1720
**AB**		Cello Concerto, C minor, RV 401, 1^st^ movement	1720s
**B) Viennese School**			
	**Composer**	**Composition**	**Year of composition**
A	Ludwig van Beethoven	String Quartet no. 1, F major, op. 18, 1^st^ movement	1799
**B**		Symphony no. 5, C minor, op. 67, 1^st^ movement	1804
**C**	Joseph Haydn	Cello Concerto, no. 1, C major, Hob. VIIb-1, 1^st^ movement	1761
**D**		String Quartet, no. 3, C major, Hob. III:77, 1^st^ movement	1796
**E**	Wolfgang Amadeus Mozart	Violin Concerto, no. 3, G major, K 216	1775
**F**		String Quartet, no. 19, C major, K 465	1785
**G**		Symphony no. 41 (Jupiter), C major, K 551, 1^st^ movement	1788
**H**		String Quartet, no. 21, D major, K 575, 1^st^ movement	1789
**I**	Franz Schubert	Symphony no. 5, B-flat major, D 485, 1^st^ movement	1816
**J**		String Quintet, C major, op. 163, D 956, 1^st^ movement	1828
**C) Romantic era**			
	**Composer**	**Composition**	**Year of composition**
**A**	Johannes Brahms	Zwei Lieder, Postillons Morgenlied, E-flat major	1874
**B**		Verlorene Jugend, D minor, op. 104, no. 4	1888
**C**	Anton Bruckner	String Quintet, F major, WAB 112, 1^st^ movement	1878
**D**		Symphony no. 7 in E major, Adagio, WAB 107	1881−83
**E**	Frédéric Chopin	Valse, A minor, B. 150	1843
**F**		Waltz ‘Minute Waltz’, D major, op. 64, no. 1	1846−47
**G**	Antonín Dvořák	Romance, F minor, op. 11, B. 39	1873
**H**		String quartet, A minor, op. 16, no. 7	1874
**I**	Edvard Grieg	Holberg Cantata, B-flat major, EG 171	1884
**J**		Valgsang, B-flat major, EG 149	1893
**K**	Gustav Mahler	Symphony no. 1, D major, 1^st^ movement ‘Ging heut’ morgen über’s Feld’	1888
**L**		Symphony no. 5, C-sharp minor, 1^st^ movement	1902
**M**	Felix Mendelssohn Bartholdy	String Quintet no. 1, A major, op. 18, 1^st^ movement	1826
**N**		Denn er hat seinen Engeln befohlen, G major, MWV B 53	1844
**O**	Sergei Prokofiev	Gavotte, F-sharp minor, op. 32, no. 3	1918
**P**		Romeo and Juliet, 3. Montagues et Capulets, G major	1935
**Q**	Sergej Rachmaninov	Symphony no. 2, G major, Adagio, 3^rd^ movement	1906
**R**		Vocalise, E minor, op. 34, no. 14	1912
**S**	Nikolai Rimsky-Korsakov	Scheherazade, Danse Orientale, A minor	1888
**T**		Flight of the bumble-bee from opera ‘The Tale of Tsar Saltan)‘, A minor	1899−1900
**U**	Robert Schumann	String Quartet no. 2, F major, op. 41, 1^st^ movement	1842
**V**		Soldiers‘ march, G major, op. 68	1884
**W**	Jean Sibelius	String Quartet, E-flat major, 1^st^ movement	1885
**X**		Valse triste, E minor, op. 44, no. 1	1903
**Y**	Richard Strauss	String Quartet, A major, op. 2, 1^st^ movement	1881
**Z**		String Sextet from Capriccio, F major, op. 85	1940
**AA**	Pyotr Ilyich Tchaikovsky	Serenade for String Orchestra, C major, op. 48, 1^st^ movement	1880
**AB**		Elegy for String Orchestra, G major, 1^st^ movement	1884
**AC**	Giuseppe Verdi	Rigoletto, La donna e mobile, B major	1851
**AD**		La Traviata, Overture, E major	1852
**AE**	Richard Wagner	Tannhäuser, Overture, E major, WWV 70	1845
**AF**		Siegfried Idyll, E-flat major, WWV 103	1870

The individual barcode sequence data of identical length, derived from the procedural RAMEAU v. 1.1 pipeline were manually aligned into a matrix (alignment) (S7A Fig) as well as combined pairs per composer before aligning (S7B Fig).

In order to gain an idea of which method and which parametrisation is most suitable for the analysis of such alignments/matrices, the three combined data sets were analysed under the average linkage methods UPGMA (Unweighted Pair Group Method with Arithmetic mean) [9], neighbor-joining [10], as well as the maximum likelihood [11] method. Neither UPGMA with DendroUPGMA (Distance coefficient: ‘Euclidean distance’, Bootstrap replicates: ‘100’) [12] nor neighbor-joining using with MAFFT (Method: ‘All of gap-free sites’; Substitution model: ‘Raw difference’; Bootstrap: ‘on’, ‘1000’) [13] provided quasi-phylogenetic topologies that matched well with the two objectives of the present study with sufficient boots trap support (data not shown).

**Fig 4:**
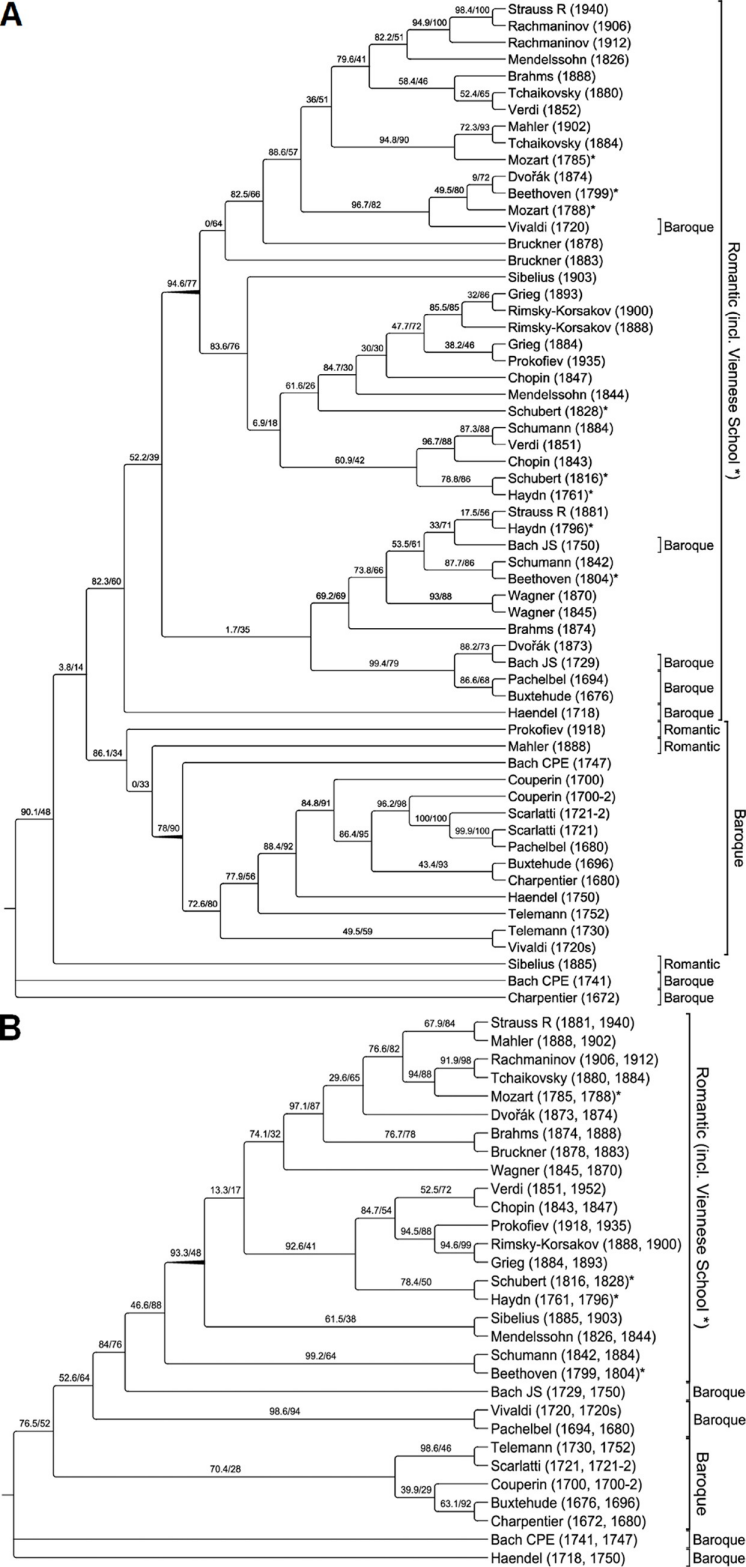
Rooted quasi-phylogeny of the Baroque era and Romantic era (including Viennese School) by maximum likelihood based on barcode sequences of *pcs* links (2-tuples of consecutive vertical pitch class sets) as elements. A. Sequences from individual works. B) Combined sequences per composer. [The alignments of the individual works are based on *pcs* cardinalities 1 to 6 (i.e. including *pcs* 1–1 to *pcs* 6–50 with 18,496 variables in individual barcode sequences and 36,992 variables in the combined sequences). The years indicate the dates of composition, and are based on the (final) year of the composition. In the case of compositions by Scarlatti and Couperin, no dates of composition could be found, so the year of the respective composer’s midlife has been chosen as a substitute. Bootstrap support values originate from ultrafast bootstrap approximation (value 1) and from SH-aLRT branch test (value 2)].

The presented quasi-phylogenies (Fig 4A and 4B) were created from S7A and S7B Fig by maximum likelihood using IQ-TREE [14] (sequence type: ‘binary’; substitution model: ‘auto-detect’; ‘ultrafast bootstrap approximation’ [15]; number of bootstrap alignments: ‘1000’; SH-aLRT branch test [16]: ‘yes’, repeats: ‘1000’). The quasi-phylogenies were visualised in the software TreeGraph [17]. The resulting topologies were rooted automatically and the clade tips sorted chronologically (‘move subtree up/down’) within the constraints of the clade topologies.

## Results

It can be shown that polyphonic compositional works of music have intrinsic structures that can be used for the structural analysis of musical works within or across particular musical eras. The method presented here derives the data directly from the musical structure itself as encoded in MIDI-formatted files, and is free of subjective influences, so that a generally usable framework for systematic comparative analyses can be built on it. This study sees itself merely as a proof of concept for a data-driven approach of the analysis of structural evolution using compositions from the Baroque to the Romantic eras (Table 1). Examples from other eras, genres or styles as impressionism, dodecaphony, modernism, rock and jazz are not included here, but could also have been used as examples. It can be shown that under maximum likelihood the structural evolution of compositions can be visualised with sufficient to very good support values. It can be seen in Fig 4A that only few cases of the two investigated compositions per composer form quasi-monophyletic or quasi-paraphyletic pairs, as is the case, for example, with the examined works by A. Bruckner, S. W. Rachmaninov, N. A. Rimsky-Korsakov, D. Scarlatti, G. P. Telemann, and R. Wagner. In contrast, the two corresponding works of composers such as J. Brahms, A. Dvořák, G. Maler, and R. Strauss show great quasi-phylogenetic distances most likely due to the time intervals between the respective works. In order to assign the composers to one of the selected eras based on a more robust tree topology, the two sequences per composer were combined by concatenation, resulting in sequences with a length of 36,992 variables (S7B Fig). Fig 4B shows clearly and with quite good support that all composers occur in the clade of their respective era, being a quasi-paraphyletic clade for the Baroque era on the one hand and, on the other, a quasi-monophyletic clade for the Romantic era including the Viennese School.

The quasi-paraphyletic Baroque era clade in this analysis and shows no remarkable structures. At least, there are no ‘lost’ composers from the Romantic era clade in it. In the Romantic era clade all selected composers of that period are covered. Interestingly works of the Viennese School are not forming a quasi-monophylum but are scattered within the Romantic era clade. Within the latter clade is more or less apparent that *pcs* diversity within each era largely increases over time, i.e. early period works have comparatively low structural diversity, while late period works relatively high structural diversity with a higher proportion of extentional chromatics, but with exceptions in both directions.

## Discussion

This methodological study merely aims to provide evidence that data-driven systematic comparisons between compositional musical works are possible by transforming data that directly form the intrinsic ‘genotypic’ structures as they exist in MIDI-formatted files. Quasi-phylogenies generated from barcode sequence matrices in this way provide insight into the quasi-phylogenetic positions between different compositional works or composers, which cannot be achieved solely based on contextual metadata. The situation is rather similar to that in evolutionary biology. There, traditional taxonomic classification allows assignment of a taxon to a particular genus or family, etc., whereas the phylogenetic representation provides information on the relative position of the individual taxa among each other. Of course, the topology, i.e. the relative positions of the contained units and clades of quasi-phylogenies in the present study is relative and dependent on the choice of compositional works (units) or parts thereof to be included, as well as on the choice of analysis method and parametrisation. However, as in evolutionary biology, the stabilisation of relationships between units and tree clades can be expected to provide an increasingly reliable basis for interpretation as more barcode sequence data are included for analysis. To ‘understand’ the topologies obtained, it is recommended to compare them with the fingerprint grid diagrams and the *pcs* frequency profiles included in the diagrams (S2–S5 Figs). The green and red coded fingerprint diagram grid cells, representing tuples with extentionally chromatic *pcs*, may be of particular interest as key factors for the development of evolutionary models for tree generation.

A global correlation between quasi-phylogenetic status and chronological assignment, as achieved in the present study, is reflected to some extent in the two trees for compositions and composers. It is rather obvious, however, that the number of barcode sequences considered in relation to compositions and composers is still too small to confirm this correlation in more detail. Nevertheless, it is regarded an encouraging result that all composers appear in the clade of their respective era (Fig 4B), but the musical works of one and the same composer do not always appear in the same subclade (Fig 4A). The latter is not necessarily irritating and would even contradict findings of traditional musicological analysis, since most composers have developed their style over time according to their personal development and the current zeitgeist. Overall it could be demonstrated that the *pcs*-tuple-based structural diversity largely developed from the early Baroque to the late Romantic era.

A detailed interpretation of the tree topologies presented here can only be made with caution, since a barcode sequence of 20 bars in length per musical work and two catenated barcode sequences per composer are definitely not sufficient to make completely reliable assumptions. Nevertheless, we assume that certain tendencies can already be outlined. In the Baroque era, the specific problem is that the inclusion or exclusion of works with ornamentation (mordents, turns, appoggiaturas etc.) can make differences in the structure of the fingerprints and consequently in the positioning of the respective barcode sequences in the quasi-phylogenetic tree. This is one of the reasons, why some of the musical works and composers from which the fingerprint grid diagrams are shown were excluded from the quasi-phylogenetic analysis.

In the Romantic era, the two barcode sequences of a composer such as R. Wagner (1845 versus 1870) show that the structural features even within a composer’s work do not necessarily increase with time (Fig 4A). In contrast, the works of J. Brahms and R. Strauss each appear in different major subclades, indicating their comparatively great compositional flexibility. The quasi-phylogenetic gap between the compositions of R. Wagner and those of A. Bruckner is remarkable, the latter in sister relationship to J. Brahms (Fig 4B). An extreme is G. Mahler, who in 1888 provided a composition with structural features close to those in the Baroque era clade and in 1902 came into quasi-phylogenetic proximity to P. I. Tchaikovsky (Fig 4A). It is not entirely surprising that compositions by composers of the Viennese School cluster together with those by composers of the Romantic period. The position of W. A. Mozart is remarkable but can be explained by the selection of compositions from his late work. Experimental analysis with his earlier works (e.g., K. 216 and K. 575) showed that these even reach into the quasi-paraphyletic sister subclades of the Baroque (data not shown).

When analysing the quasi-phylograms, the question arises as to what exactly these clades represent. Since the data basis of the quasi-phylogenetic trees is barcode sequences consisting of *pcs* 2-tuples, the characteristic features of the various clades represent specific combinations of *pcs* 2-tuples. These in turn, are determined by the ‘cardinality’ of the *pcs* in the sense of Forte [5] (not to be confused with the number of voices) and/or the abundance of *pcs* belonging to extentionally diatonic and extentionally chromatic chords or vertical pitch sets in the sense of Rambold [6]. It is apparent that the compositions positioned near the root, tend to have *pcs* of a lower cardinality and/or a lower proportion of extentionally chromatic vertical pitch sets. In the late Romantic era, the situation is reversed, with composers whose musical oeuvres tend to have a higher *pcs* cardinality and/or a higher proportion of extentionally chromatic pitch sets.

It remains to be seen whether a higher number of compositions or elements considered in the alignments really leads to better robustness of the quasi-phylogenetic tree topologies. However, in well-planned music analysis projects, i.e. those based on a specific concept, it may happen that only a limited number of compositional works or parts thereof, need to be examined anyway. From the observations we have made in this study (quasi-phylogeny of compositions versus that of composers with combined sequences), it seems fairly certain that merging barcode sequences from different compositions or from movements of a given piece of music will lead to more meaningful and better-supported quasi-phylogenetic topologies. Moreover, focusing on the works of one composer might show how widely the composer’s oeuvre is scattered across the quasi-phylogeny of music.

Before the method of analysis presented here can be widely applied, it is likely that a collaborative process will take place in the research community over time to reach agreement on an optimal approach to object selection criteria. This concerns, among other aspects, the number of variables per barcode sequence and the minimum sequence number to be included in an analysis. In composition-based analysis, there may be a convention that each movement of a given musical work should be represented by at least one barcode sequence, analogous to multi-gene approaches in evolutionary biology. Of course, it would be also conceivable to create and combine sequences of whole movements analogous to full genome sequencing. A particular challenge may be the question of ornamentation as in Baroque music. In this case, it is possible to remove such ornamental structures before the MIDI files are created for subsequent analysis. Furthermore, the inclusion of specific evolutionary models [18] could provide further improvement of the results. Their development could be a task for future music informatics. In addition, the extent to which branch support and branch lengths need to be taken into account will need to be discussed [19], and the same applies to bootstrap replicate numbers [20]. Different approaches could be developed to analyse the similarity of the compositional oeuvres, either on the basis of combined barcode sequences from different compositional works (by concatenation as in the sequence alignment for Fig 4B, or by creating alignments of consensus sequences) or based on individual compositions or parts thereof, and which method/algorithm and parameterisation should be chosen.

We assume that in future studies of this kind, in which perhaps hundreds of music compositional works will be examined, more or less large deviations of the quasi-phylogenetic tree topologies presented here will emerge, but that sooner or later they will stabilise through saturation and form a kind of framework for musicological work. Certainly, a musicology based solely on such analyses would be rather poor in substance. But musicological analyses that do not take these intrinsic structures into account are arguably incomplete. It should be emphasised again that in this approach, the basic analytical framework (*pcs*-tuple-based grid diagrams and quasi-phylogenetic trees derived from them) is entirely based on the intrinsic structures of the targeted compositional works, and not only on accompanying contextual data (metadata) whose underlying classifications themselves already represent a kind of pre-interpretation of the features. However, by mapping such (meta-)data onto the obtained quasi-phylogenetic tree topologies, new relationships and correlations can be identified for further analysis. It should also be emphasised that the proposed approach (or other types of visualisations such as ordination diagrams based on the presented type of datasets) can be used not only for comparing works of different composers within a particular era or genre, but of course also for investigating developments within the oeuvre of a single composer.

To this end, a public repository would be useful to make data resources freely available for musicological analysis. Such infrastructure already exists in the natural sciences where public repositories exist, e.g. for genomic and proteomic sequence data such as BOLD (https://www.boldsystems.org/), GenBank (https://www.ncbi.nlm.nih.gov/genbank/) or DDBJ (https://www.ddbj.nig.ac.jp/index-e.html) for gene sequence data and UniProtKB (https://www.uniprot.org/) for protein sequence data. In order to collaboratively realise a growing number of quasi-phylogenetic trees of musical compositions, it is recommended to set up freely accessible archives in which MIDI v. 1 files are published and archived according to FAIR guiding principles [21] together with a mandatory minimum set of contextual data (metadata) for analysis, such as by means of RAMEAU v. 1.1 (online service pending) or comparable software (e.g. R scripts pending setup; https://www.r-project.org/).

## Conclusion

With the present study, we were able to show that pcs 2-tuple-based barcode sequences represent elementary ‘genotypic’ structures, which can be used to characterise of polyphonic music on the basis of fingerprints and to classify it by quasi-phylogenetic trees. By applying the maximum likelihood method, we were able to more or less unambiguously achieve the two goals of the study that a) the compositions and/or composers of at least one era formed a quasi-monophyletic group and that b) within one quasi-monophylum it was possible to read to some extent a chronology of the compositions with sufficient statistical support.

Based on experience with evolutionary analyses and visualisations in other scientific fields, we anticipate that the use of fingerprints, barcode sequences and quasi-phylogenies provides options for comparative computational and machine learning-based musicology, particularly as a structural backbone to address different aspects of music theory on the background of data from traditional and other approaches in computational musicology.

## Supporting information

S1 Fig Multi-track MIDI input files of the examined music compositional works. (ZIP)

S2 Fig Figs A−AB. Fingerprint grid diagrams of *pcs*_n_- *pcs*_n+1_ 2-tuples and relative *pcs* abundances of the examined music compositional works from the Baroque era. (ZIP)

S3 Fig Figs A−J. Fingerprint grid diagrams of *pcs*_n_- *pcs*_n+1_ 2-tuples and relative *pcs* abundances of the examined music compositional works from the Viennese School. (ZIP)

S4 Fig Figs A−AF. Fingerprint grid diagrams of *pcs*_n_- *pcs*_n+1_ 2-tuples and relative *pcs* abundances of the examined music compositional works from the Romantic era. (ZIP)

S5 Fig Fingerprint grid diagrams of the examined music compositional works from the Baroque to the Romantic era (pdf). (DOCX)

S6 Fig RAMEAU v.1 intermediate files of the examined music compositional works, including extracted from them Vertical Pitch Set files (*.vps), Rich Data Listing files (*.rdl), Pitch Class Set Listing files (*.pcs). (ZIP)

S7 Fig Alignments of A. Individual (‘compositions’) and B. Combined (‘composers’) Barcode Sequence files (*.fas) of the examined music compositional works from the Baroque, Viennese School and the Romantic era.
(ZIP)
