# Supplementary material for: Fingerprints, barcode sequences and quasi-phylogenies–Tools for analysing polyphonic music
Source: PLoS One. 2023 Mar 2;18(3):e0280478. doi: 10.1371/journal.pone.0280478 (PMC9980773; doi:10.1371/journal.pone.0280478)
Supplement: S5 Fig — (DOCX) [file pone.0280478.s005.docx]

**S5: Fingerprint grid diagrams of the examined music compositional works from the Baroque to the Romantic era (pdf).**

**[S2] Figs A−AB. Fingerprint grid diagrams of pcs n- pcs n+1 2-tuples and relative pcs abundances of the examined music compositional works from the Baroque era.**  [For details see Table 1A]
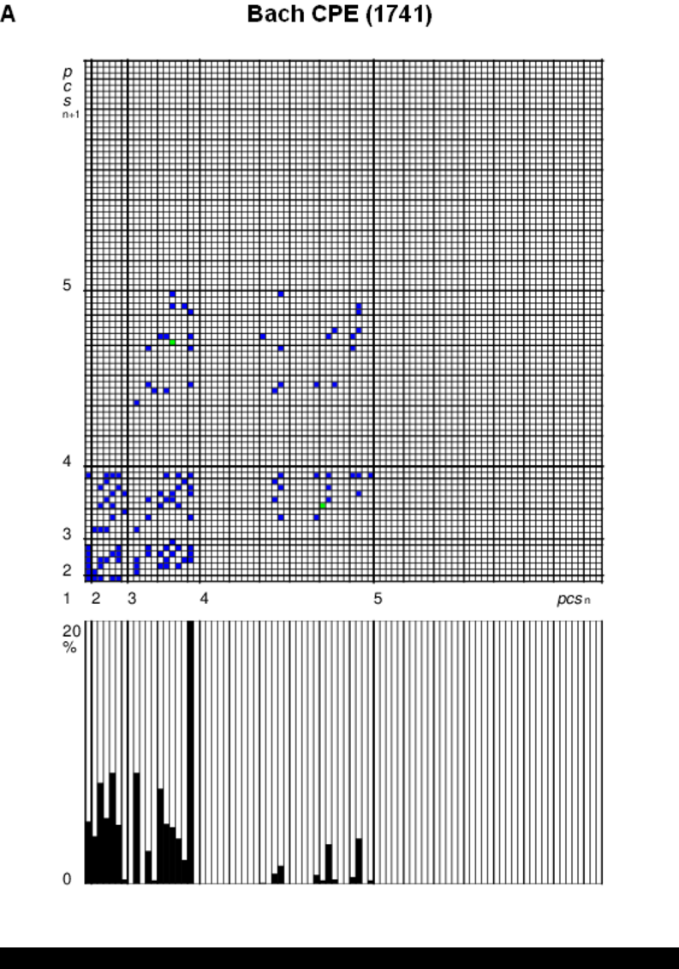

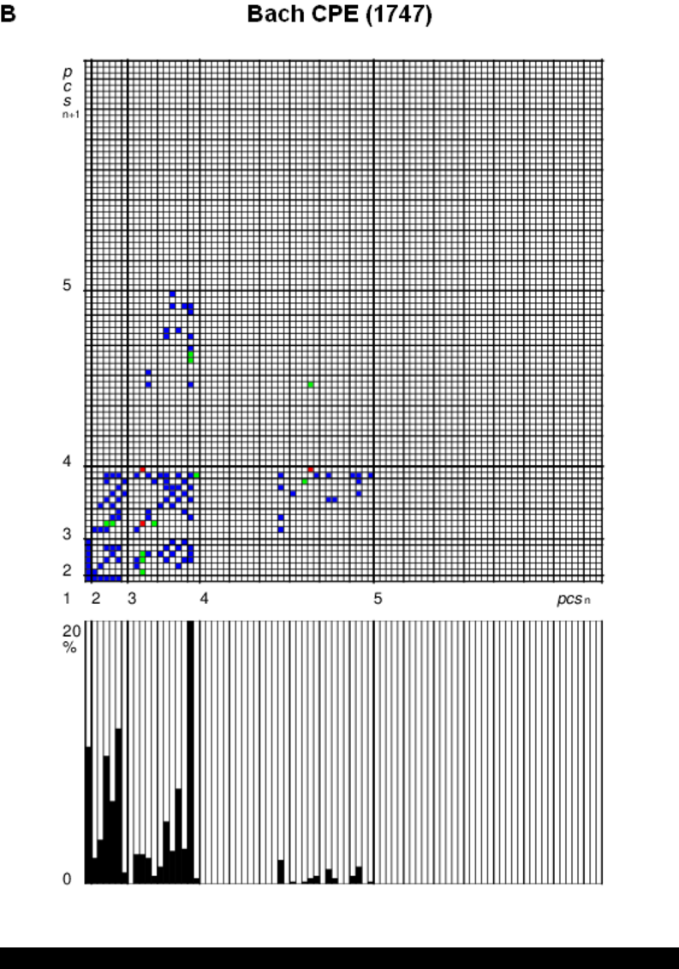

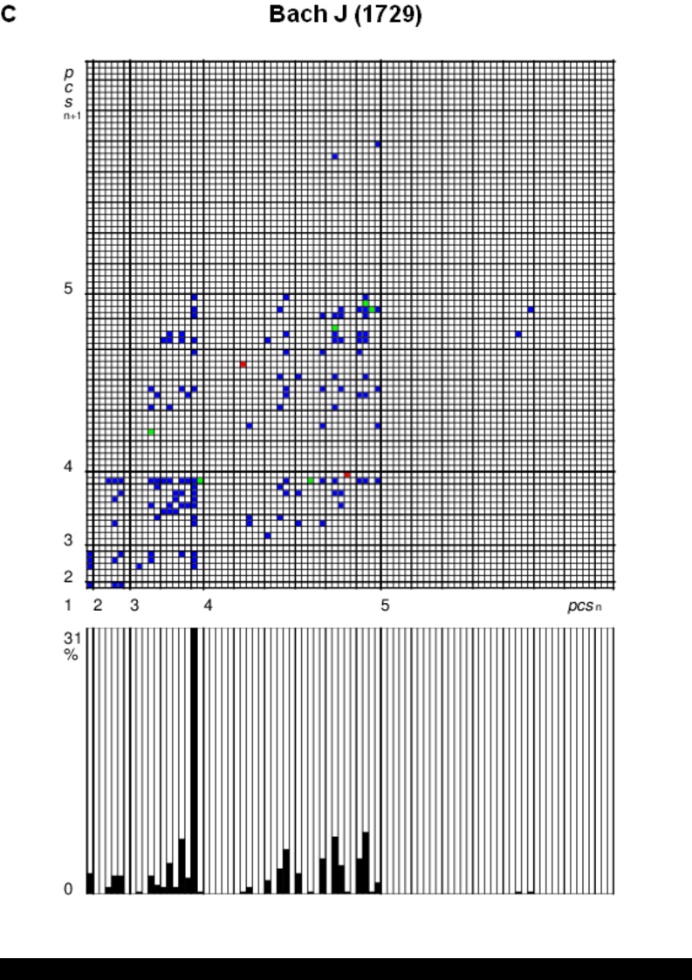

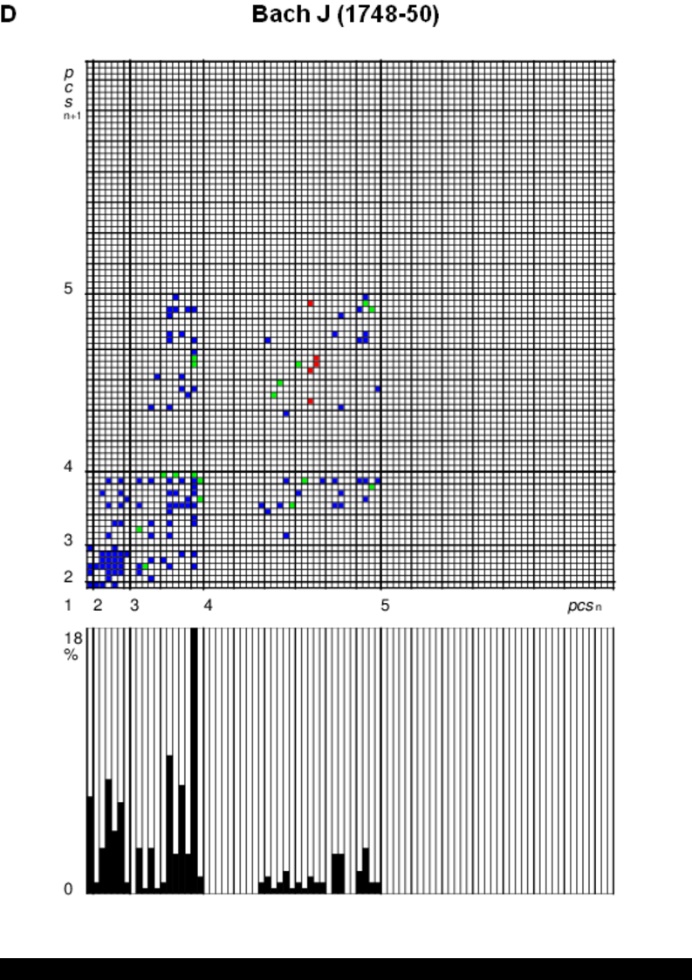


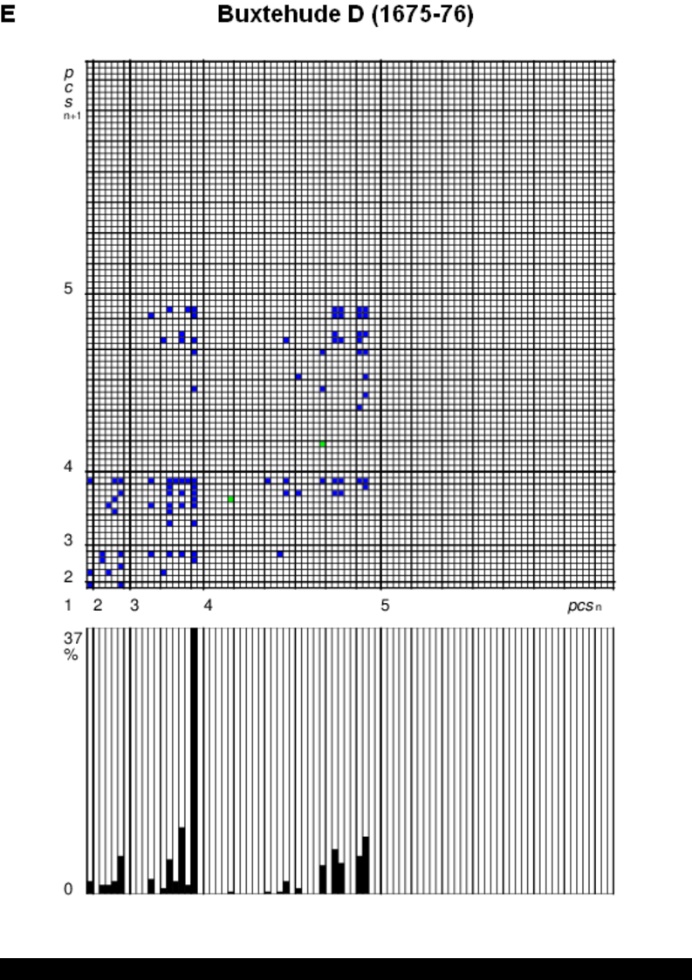

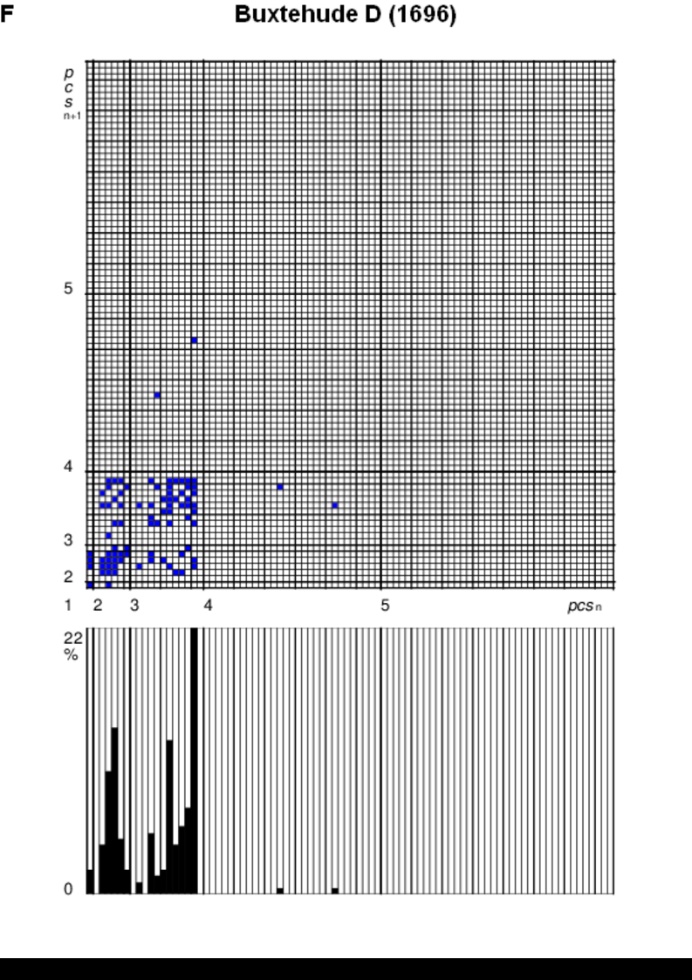
**
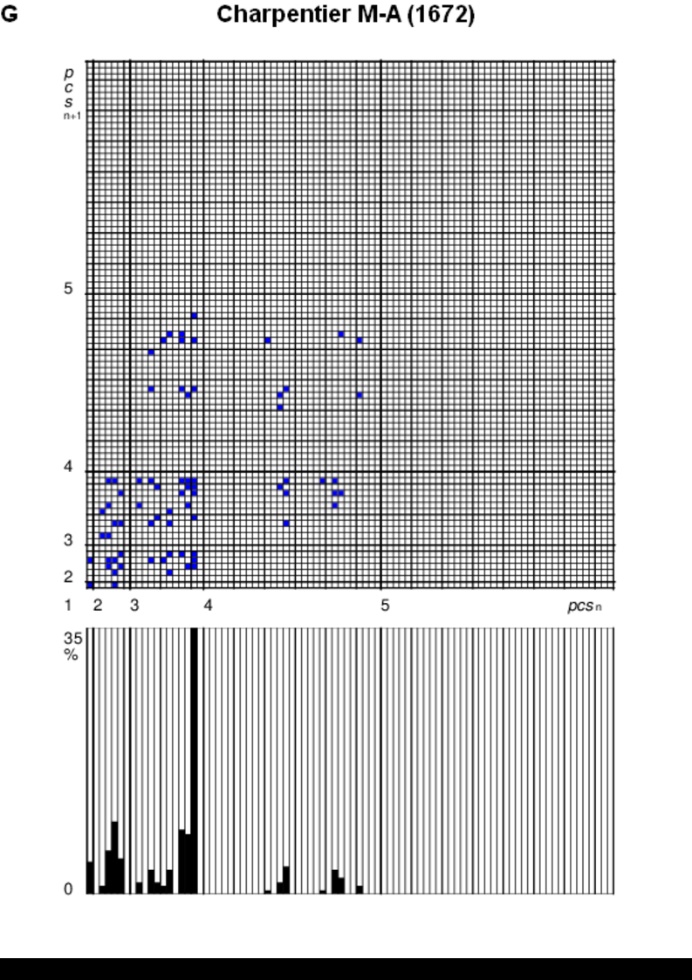

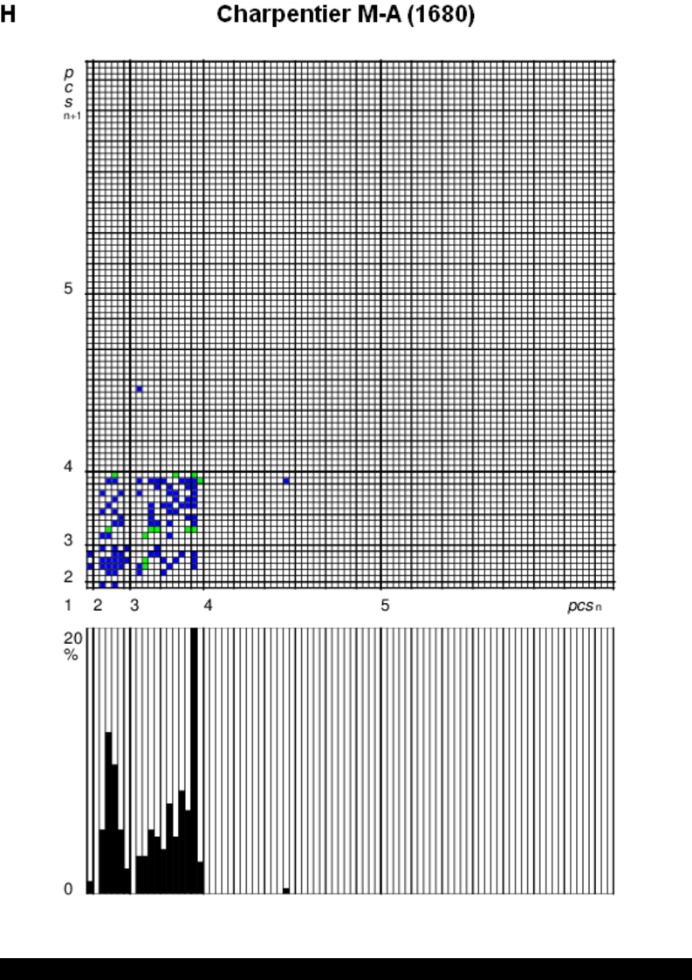

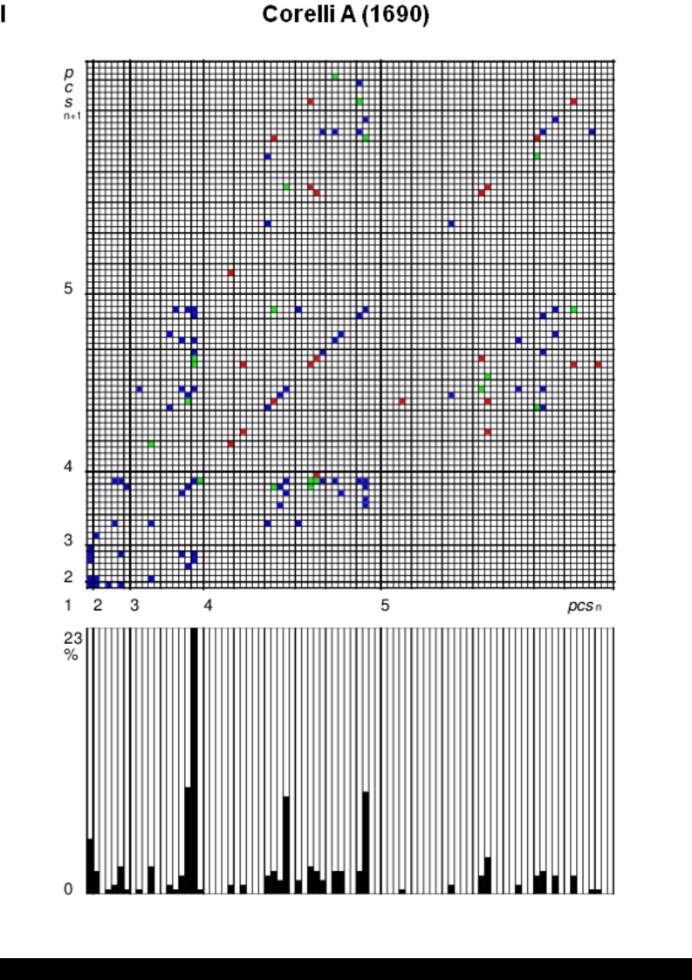

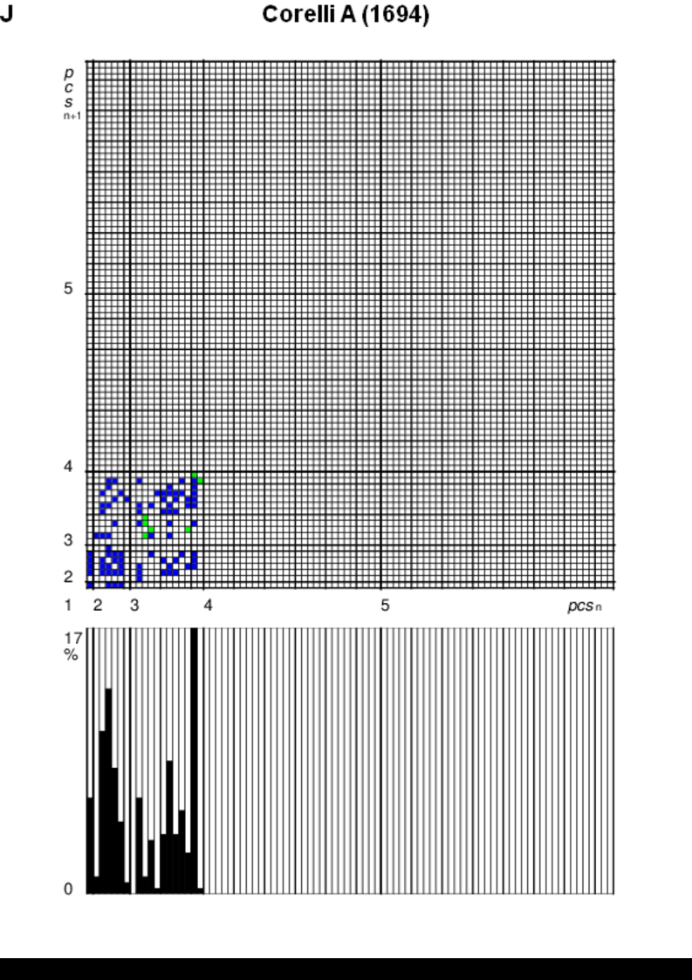

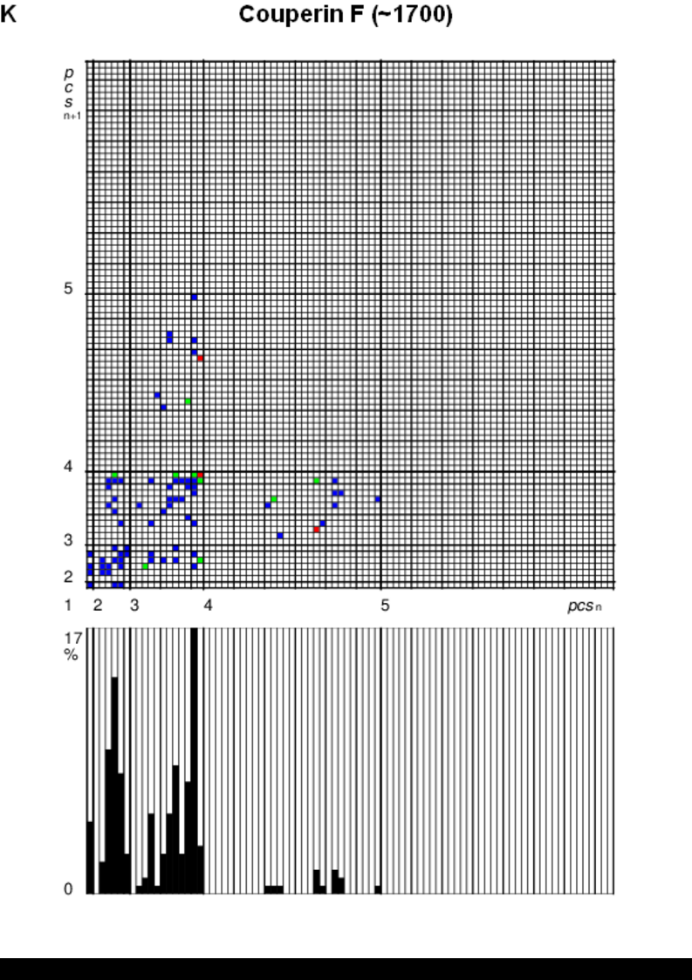

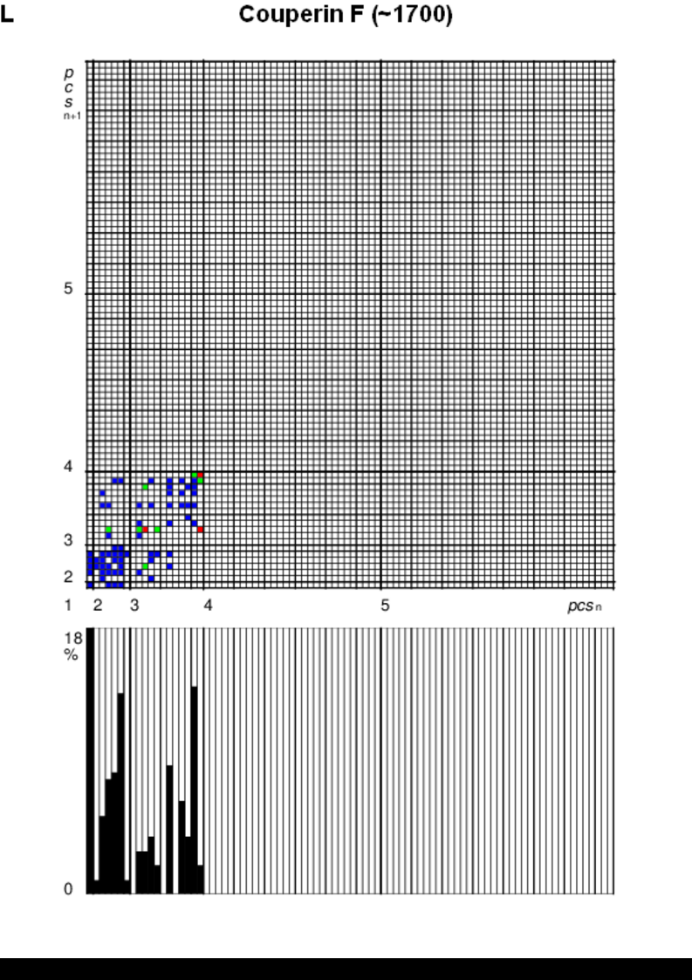

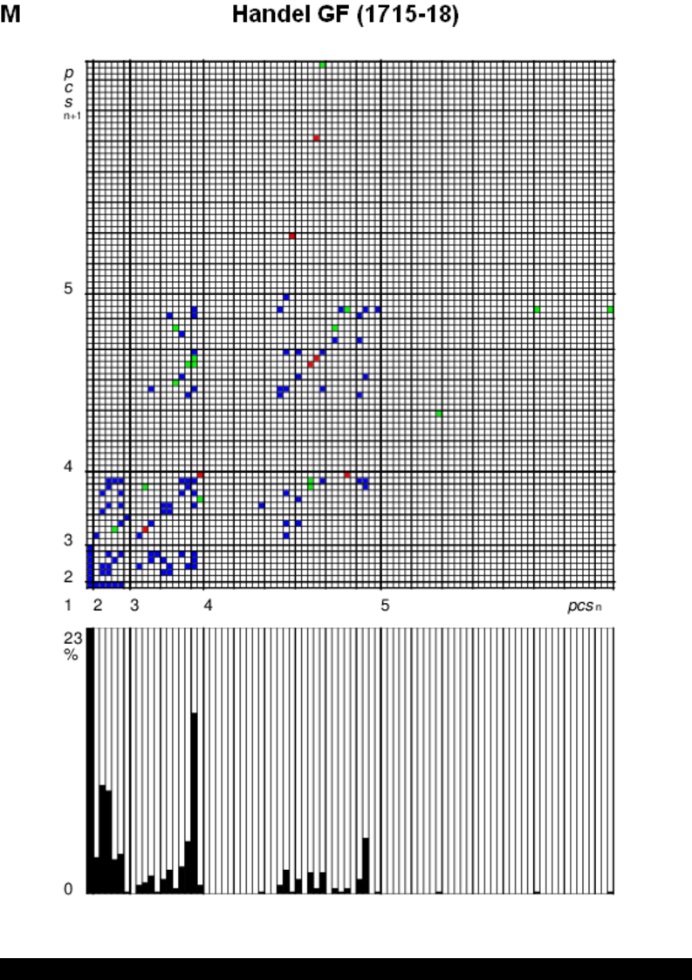

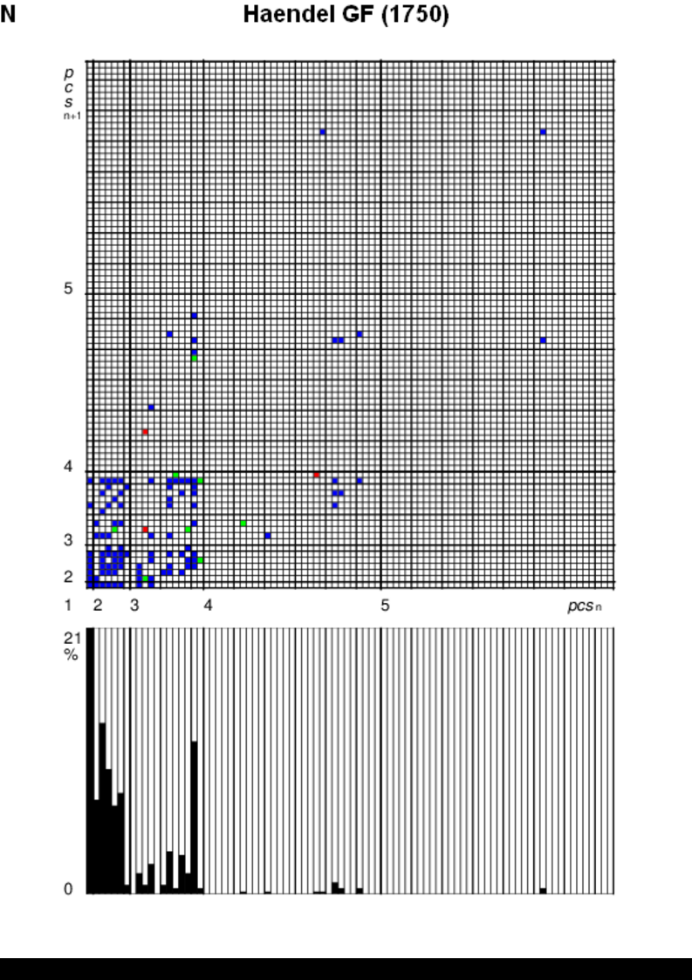

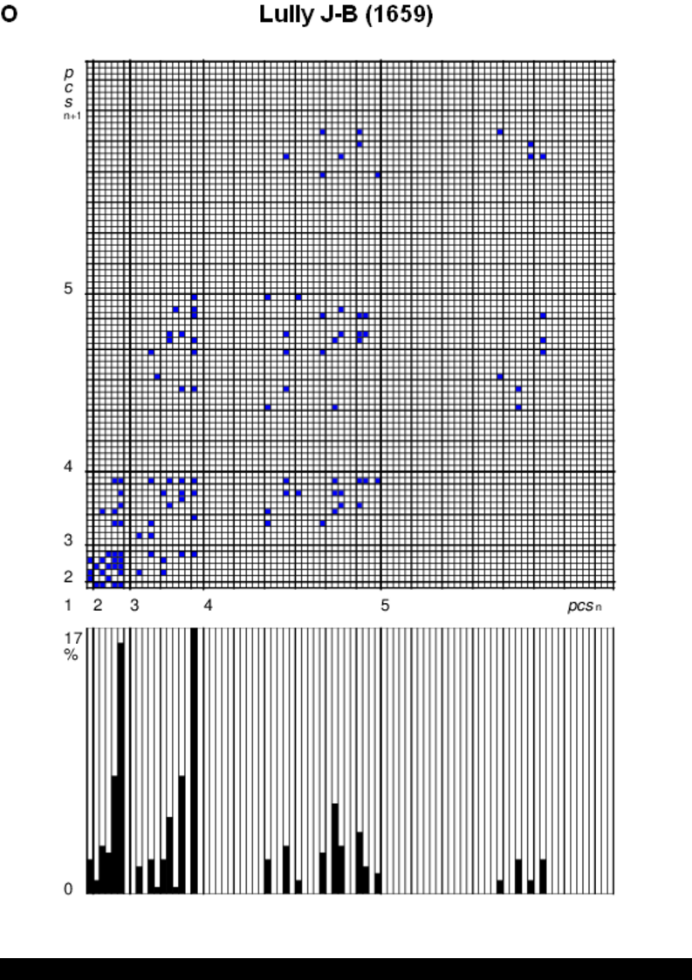

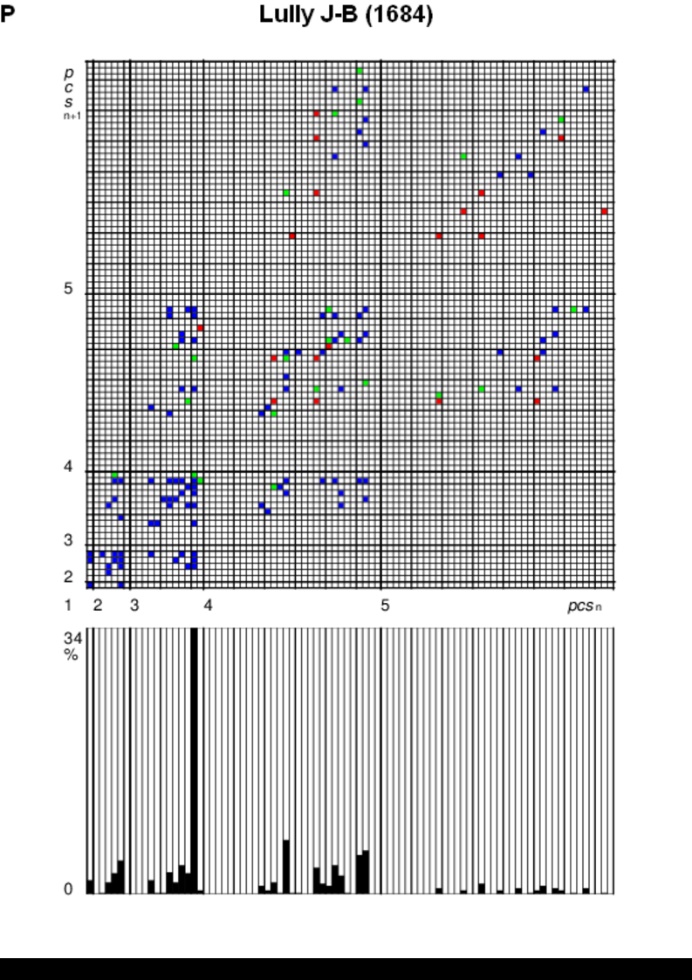

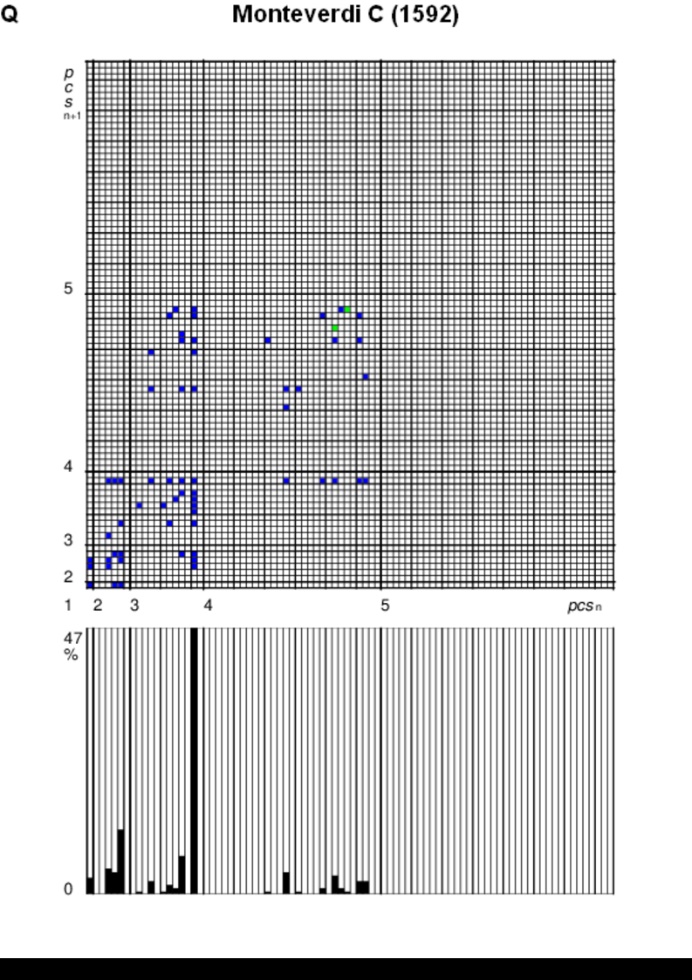

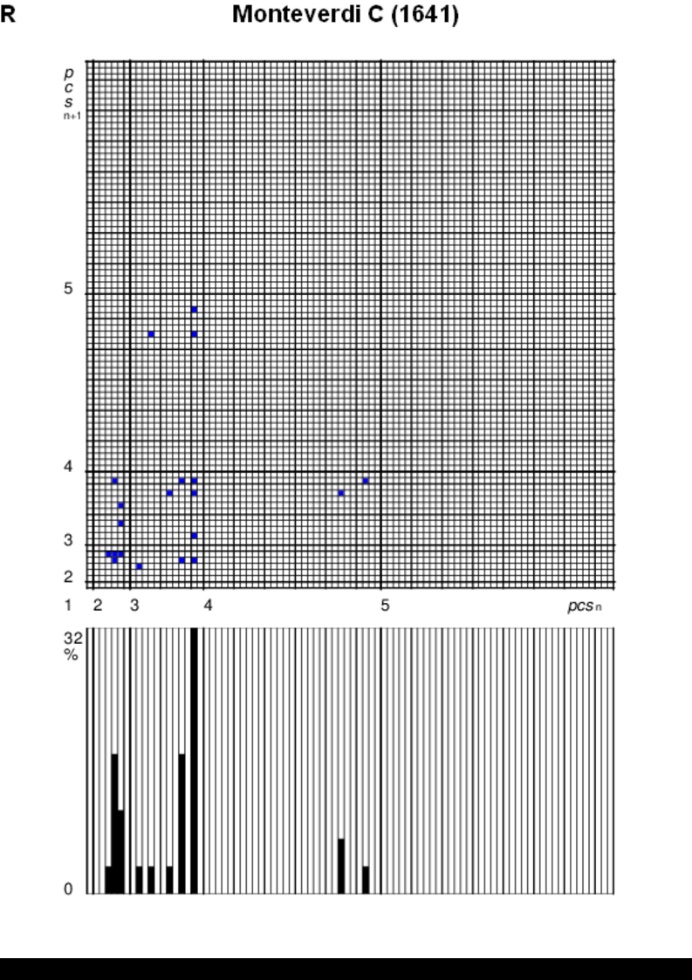

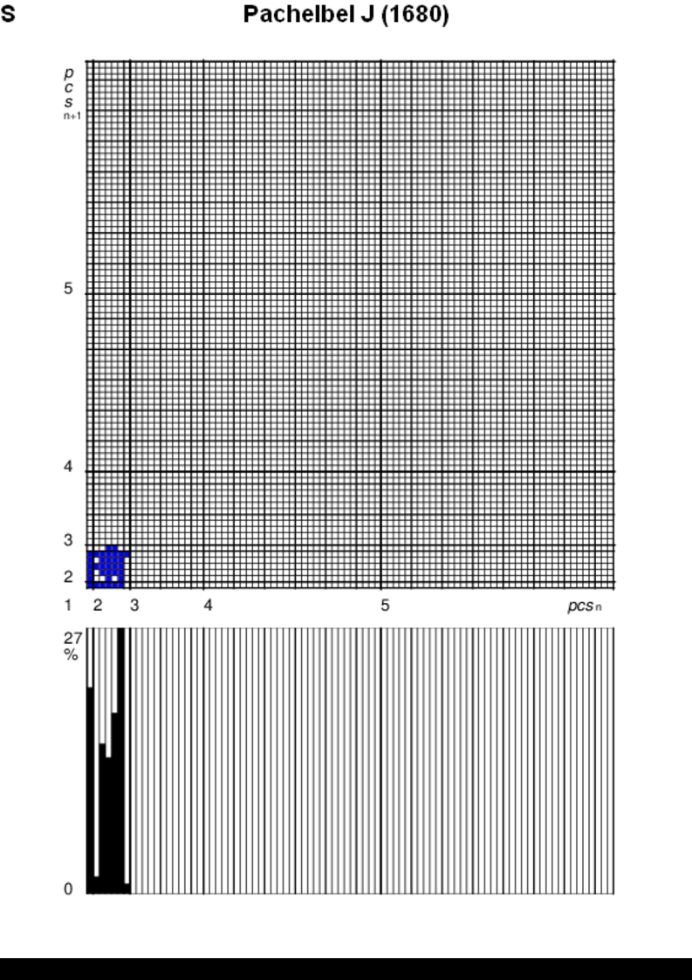

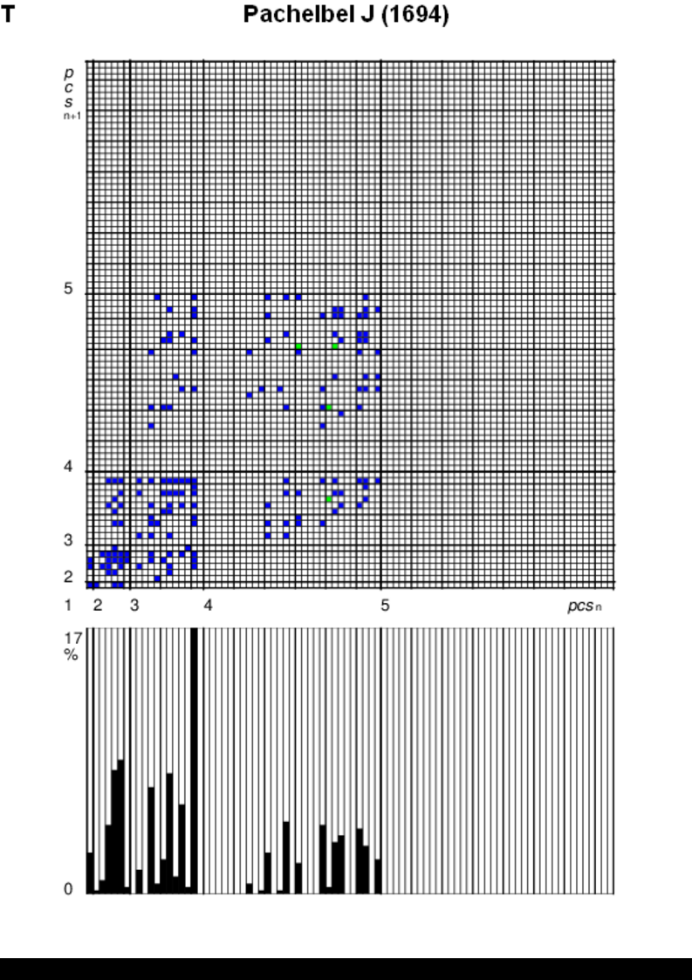

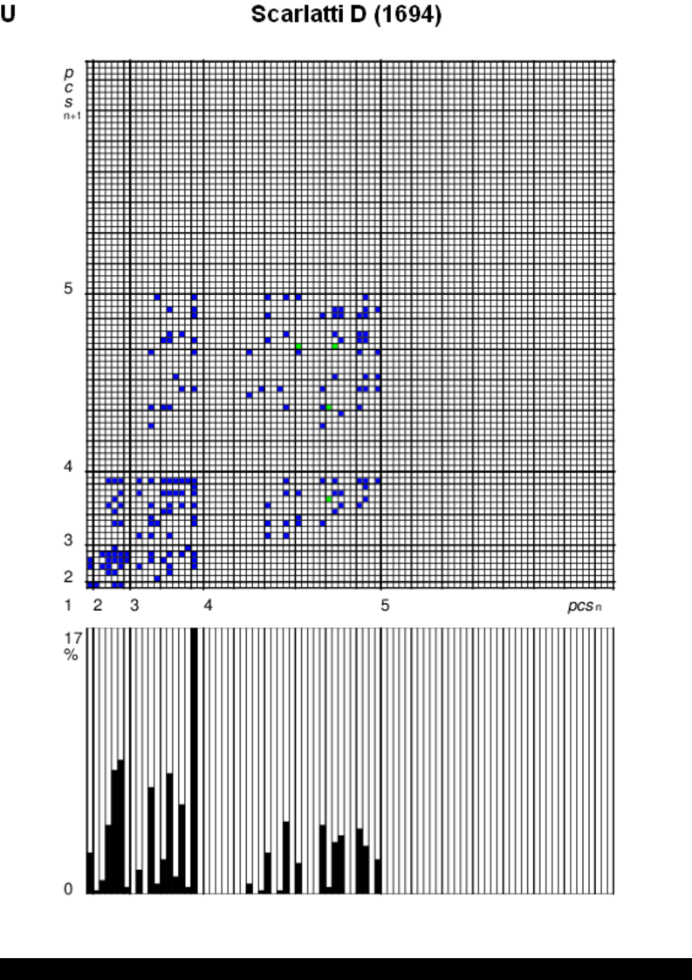

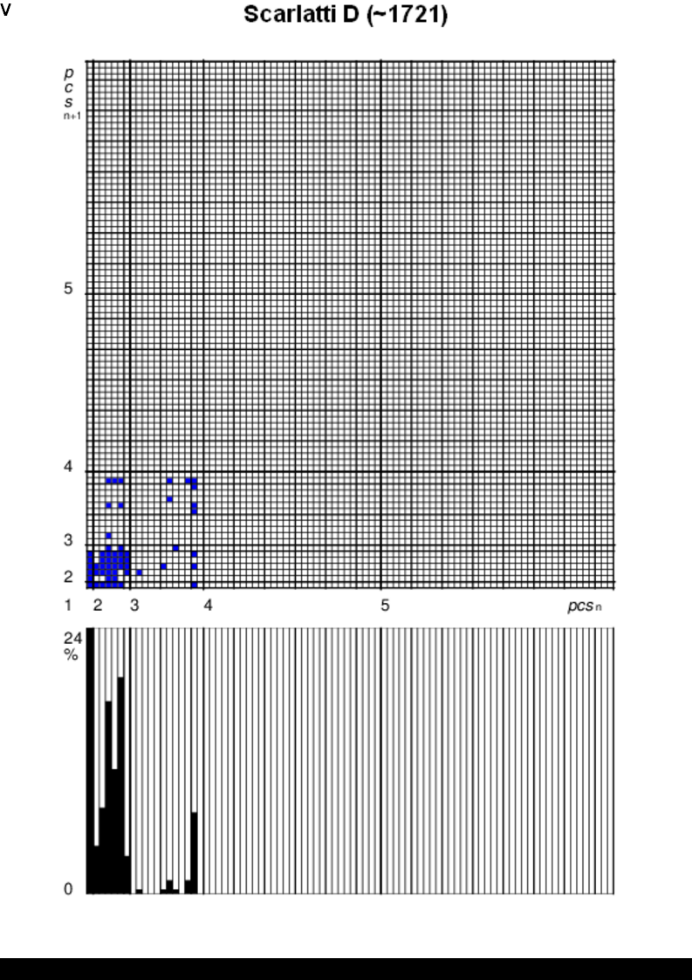

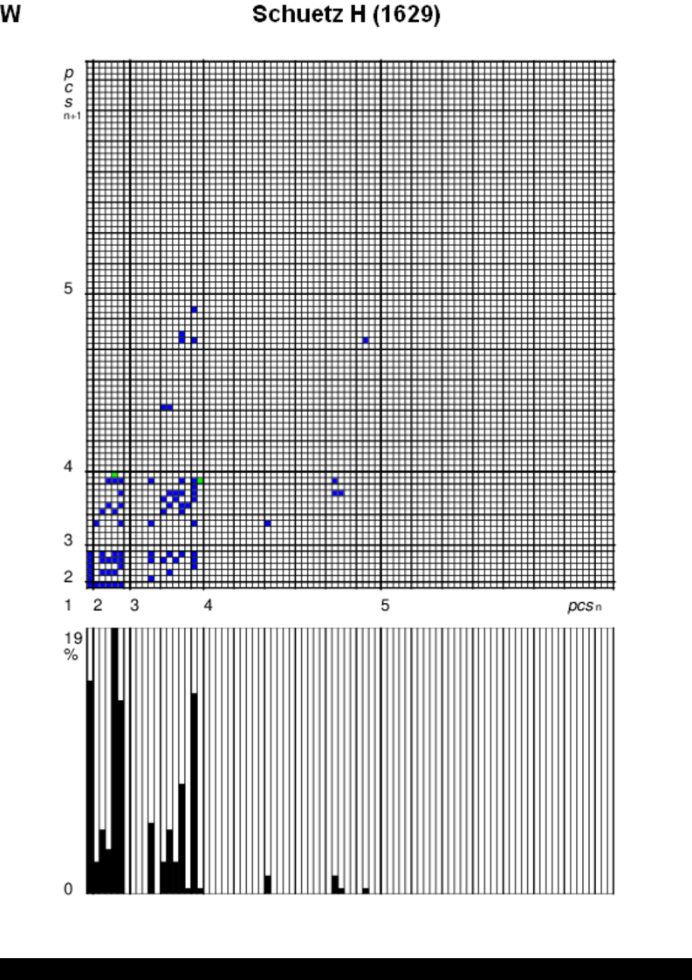

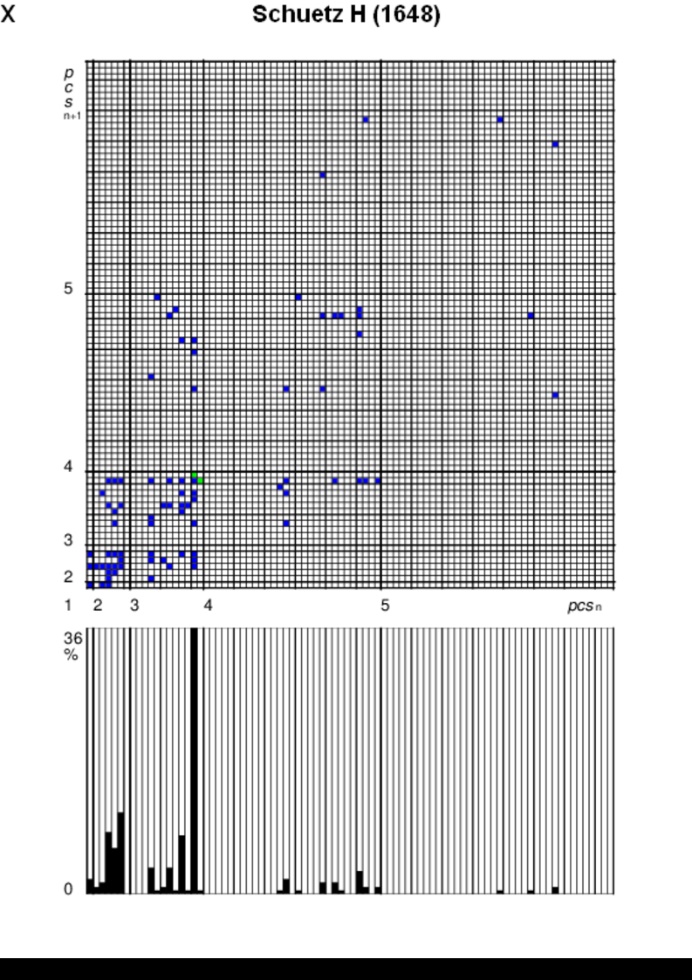

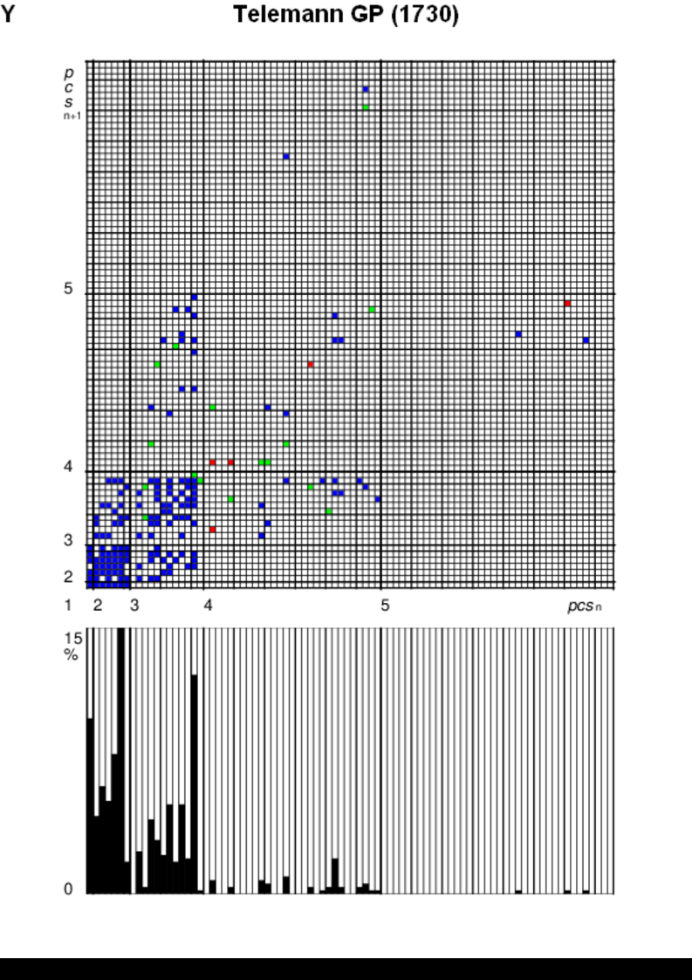

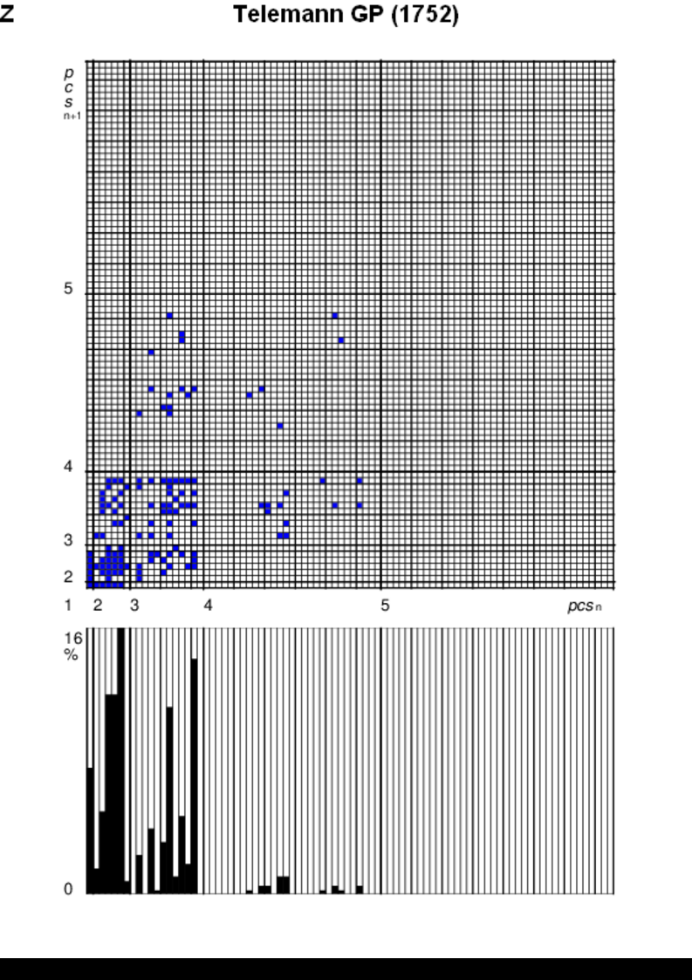

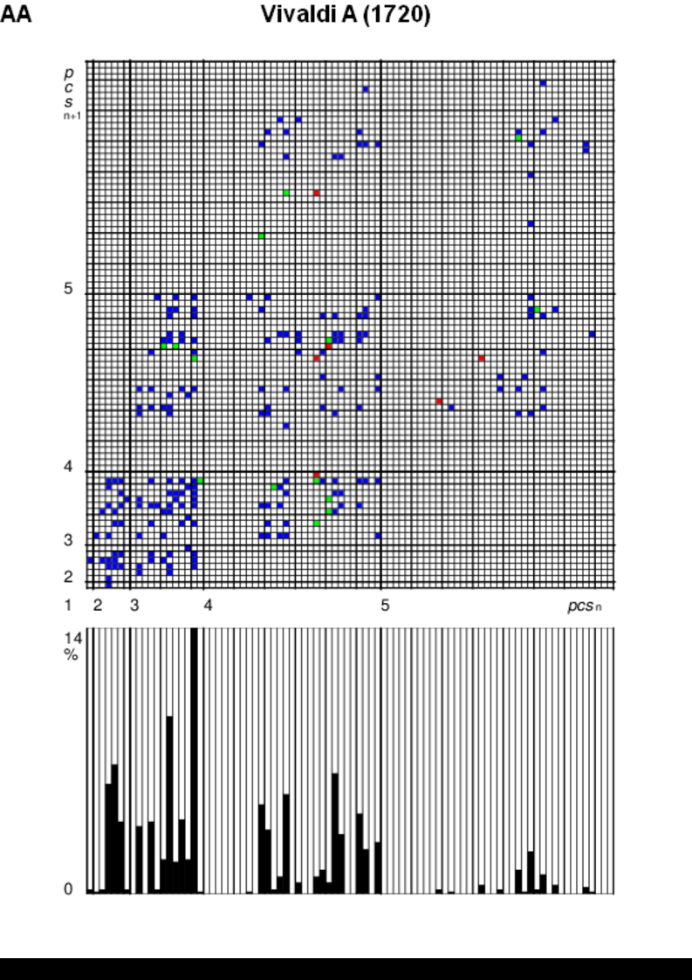

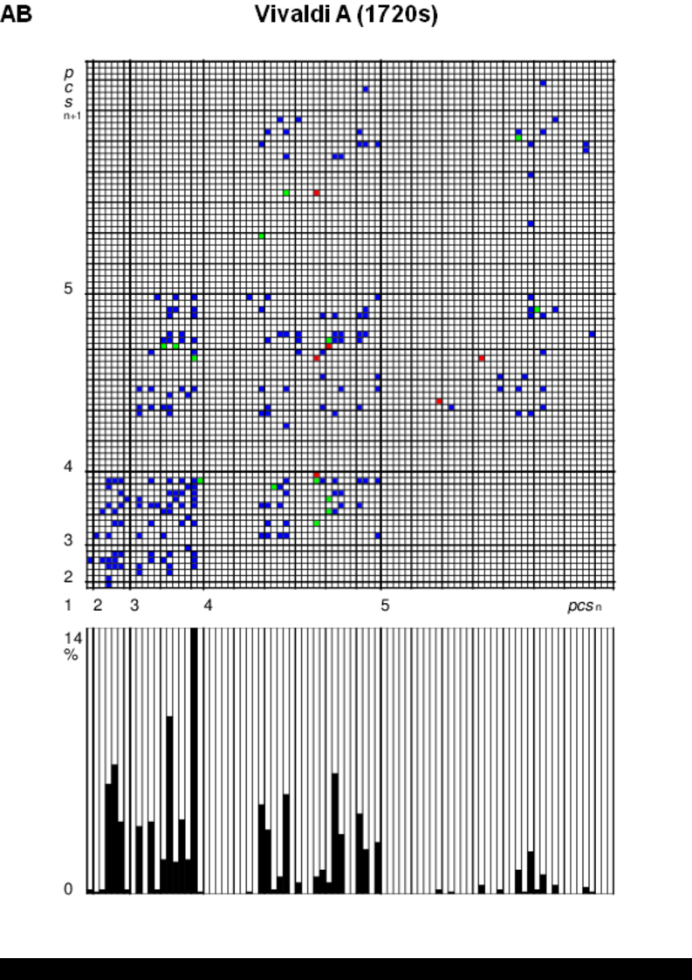
**

**[S3] Figs A−J. Fingerprint grid diagrams of pcs n- pcs n+1 2-tuples and relative pcs abundances of the examined music compositional works from the Viennese School.** [For details see Table 1B]

**
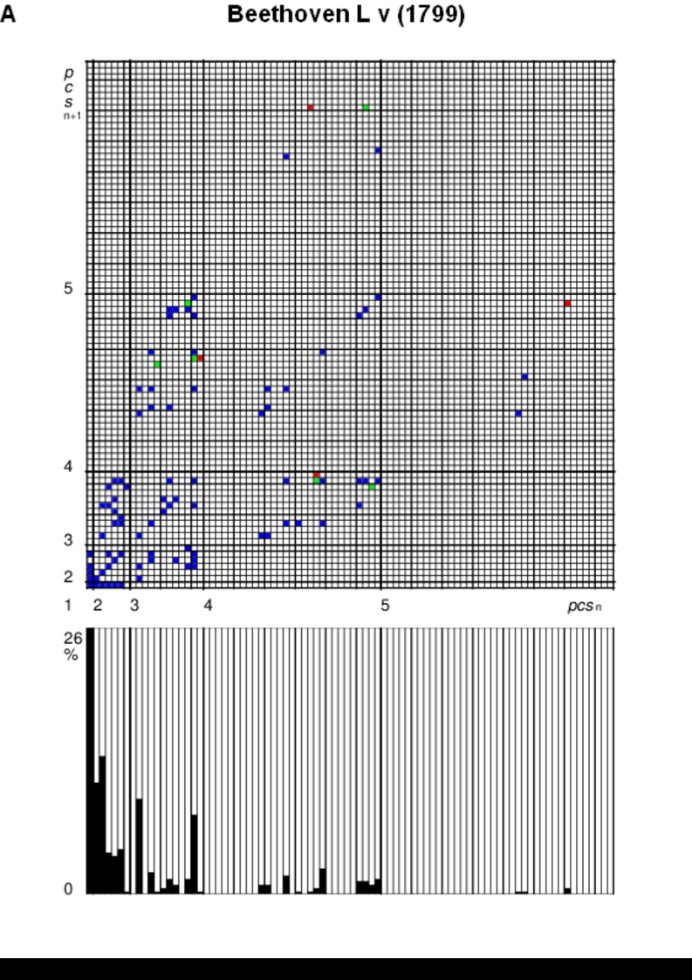

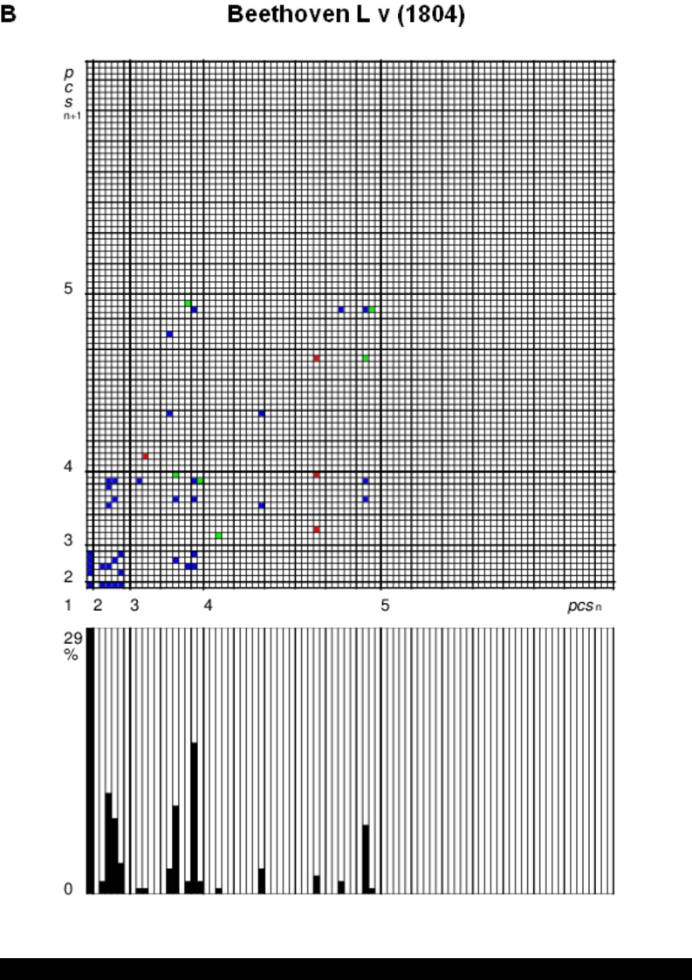

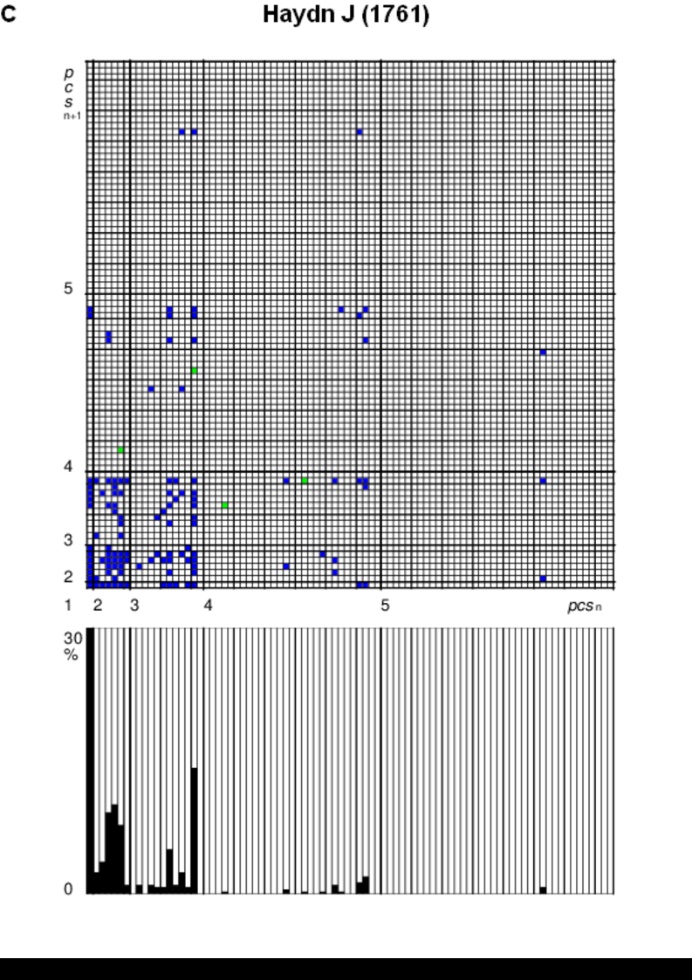

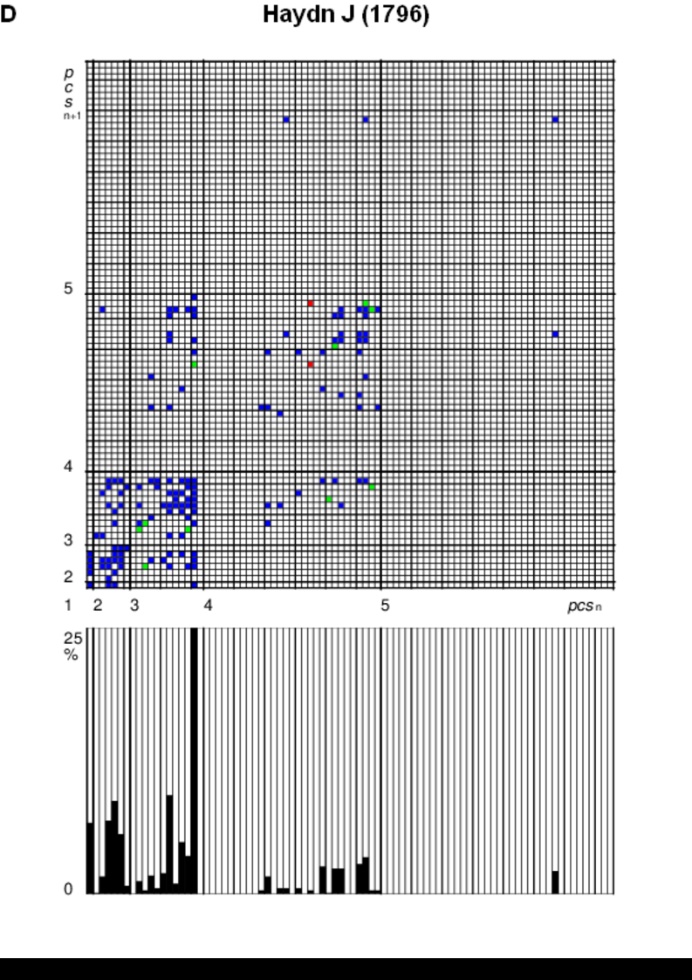

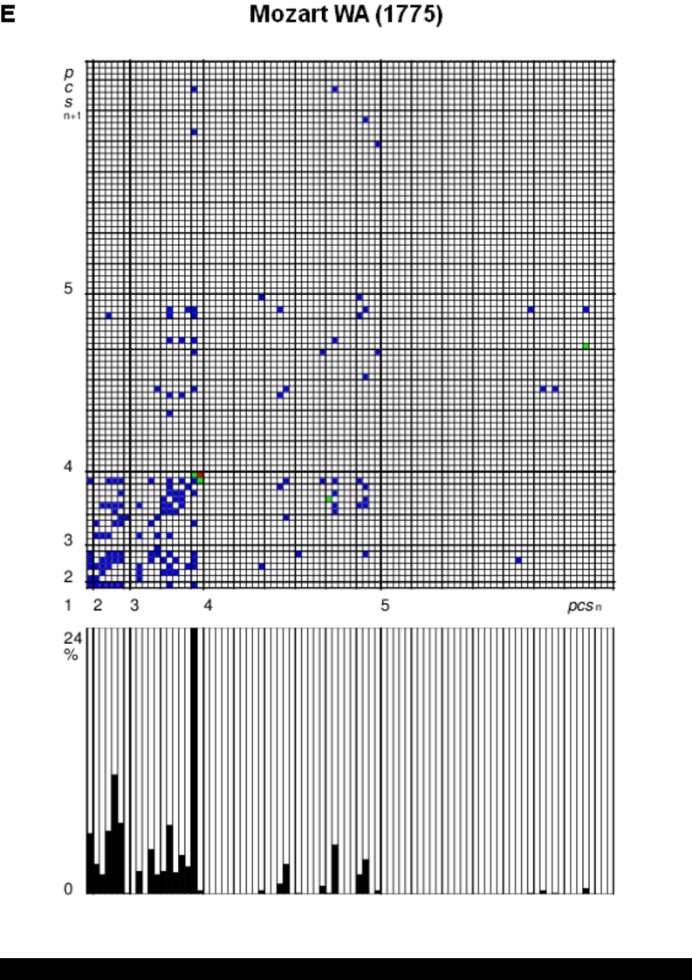

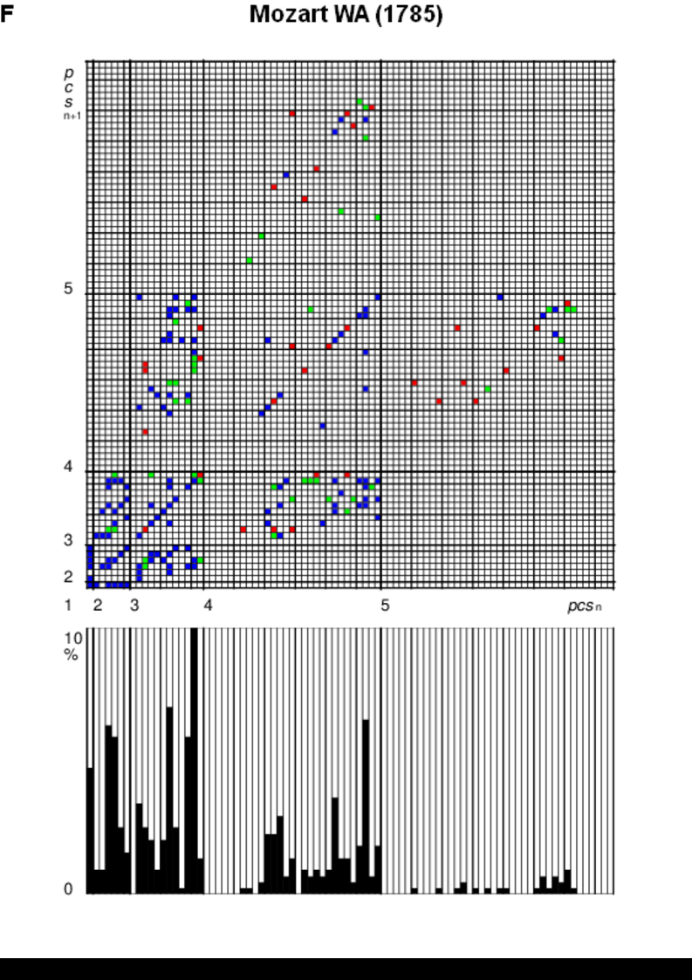

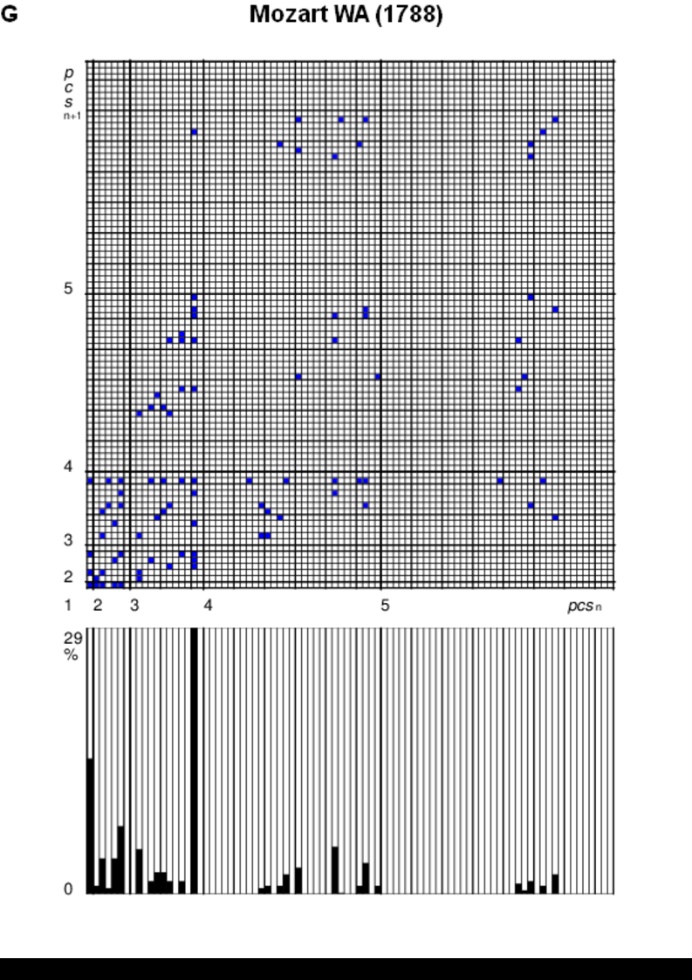

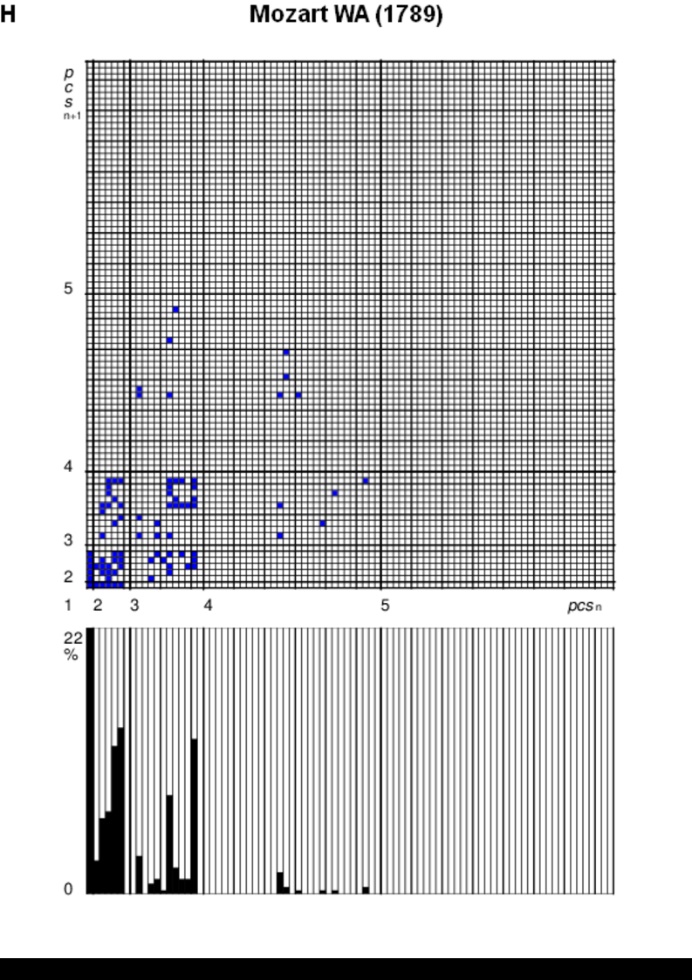

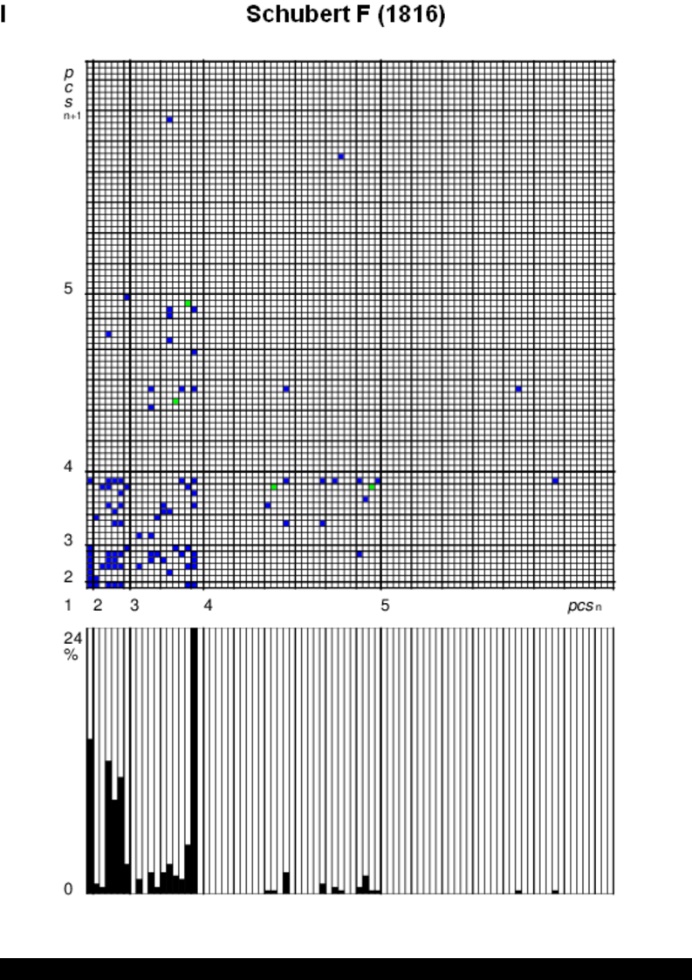

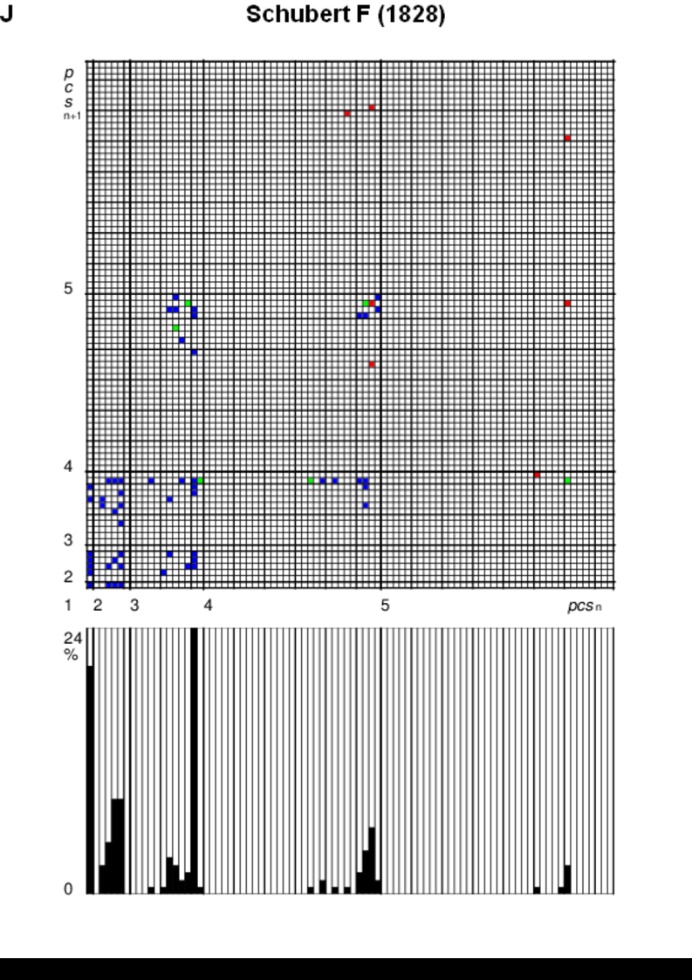
**

**[S4] Figs A−AF. Fingerprint grid diagrams of pcs n- pcs n+1 2-tuples and relative pcs abundances of the examined music compositional works from the Romantic era.** [For details see Table 1C]

**
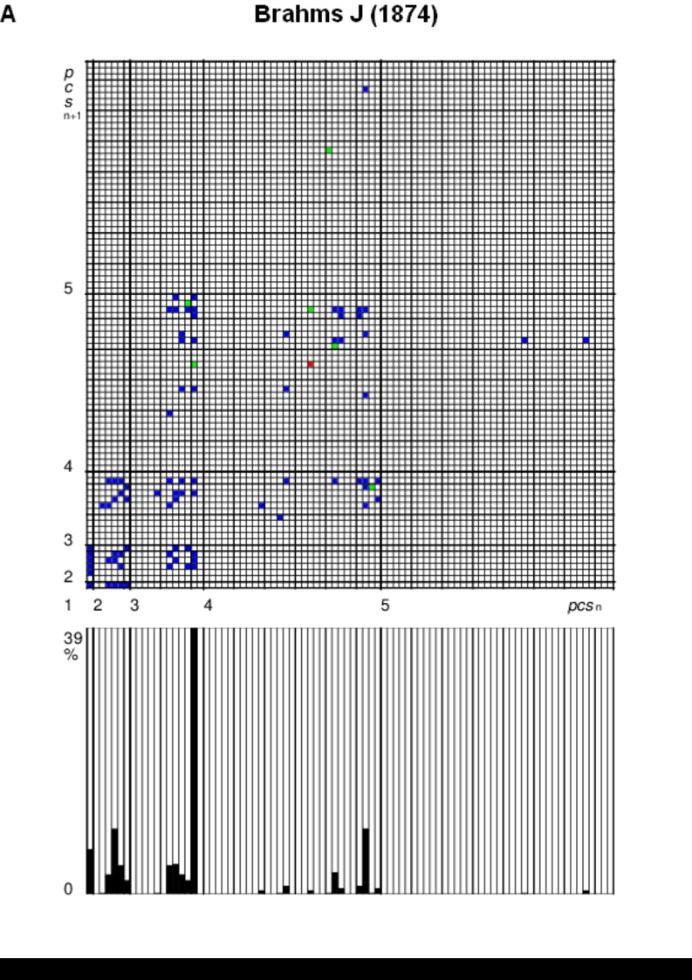

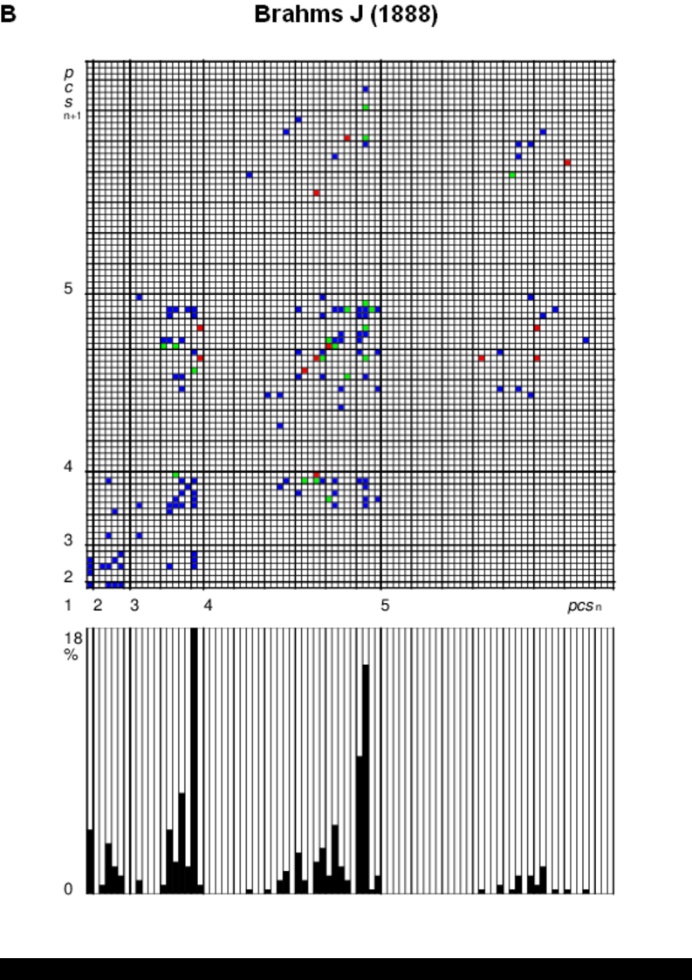

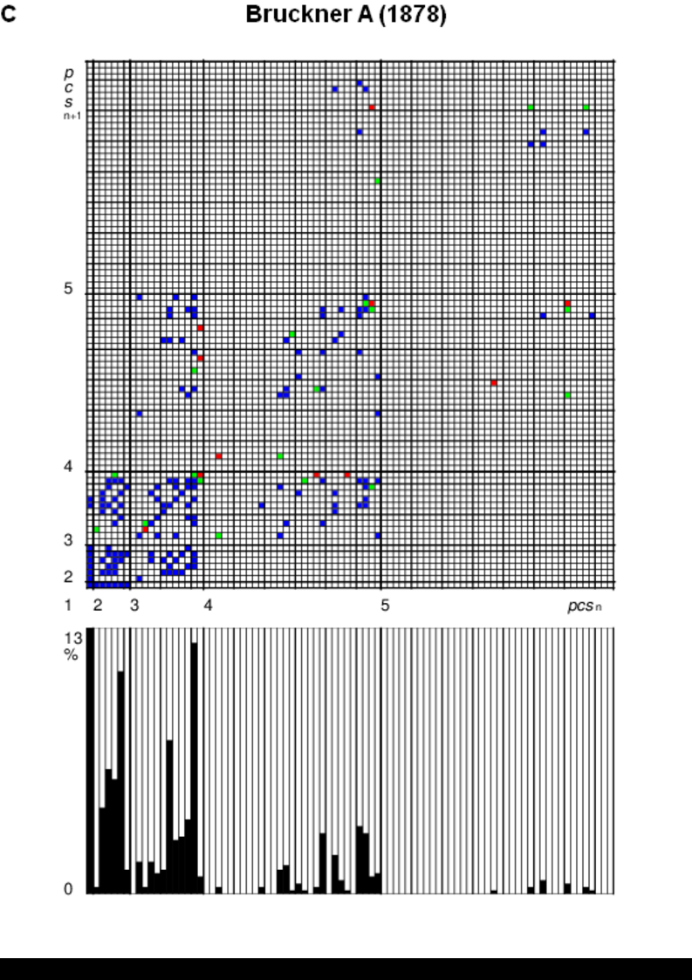

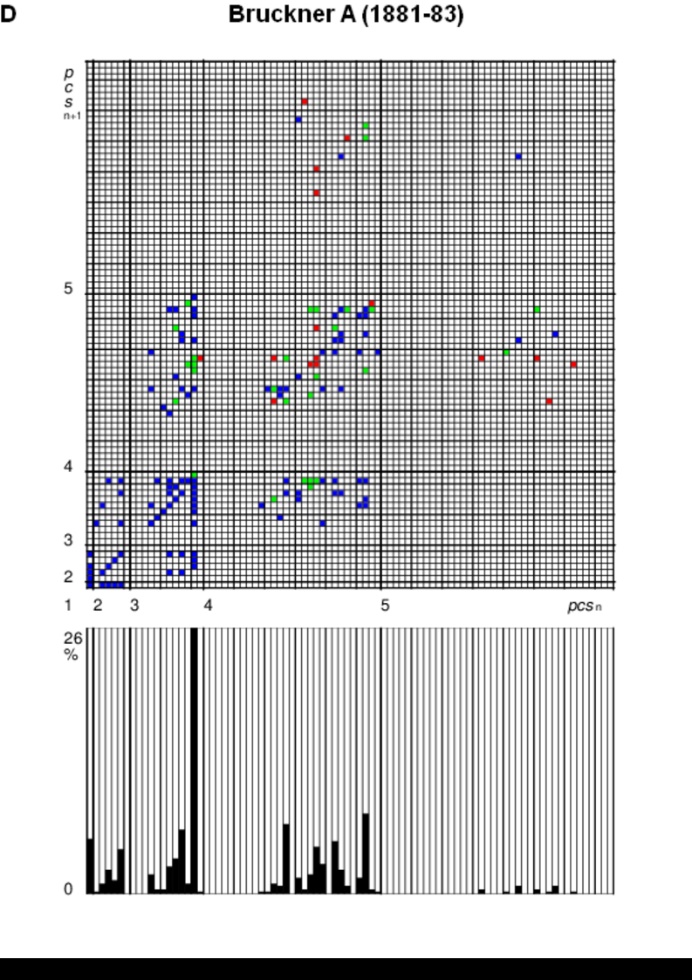

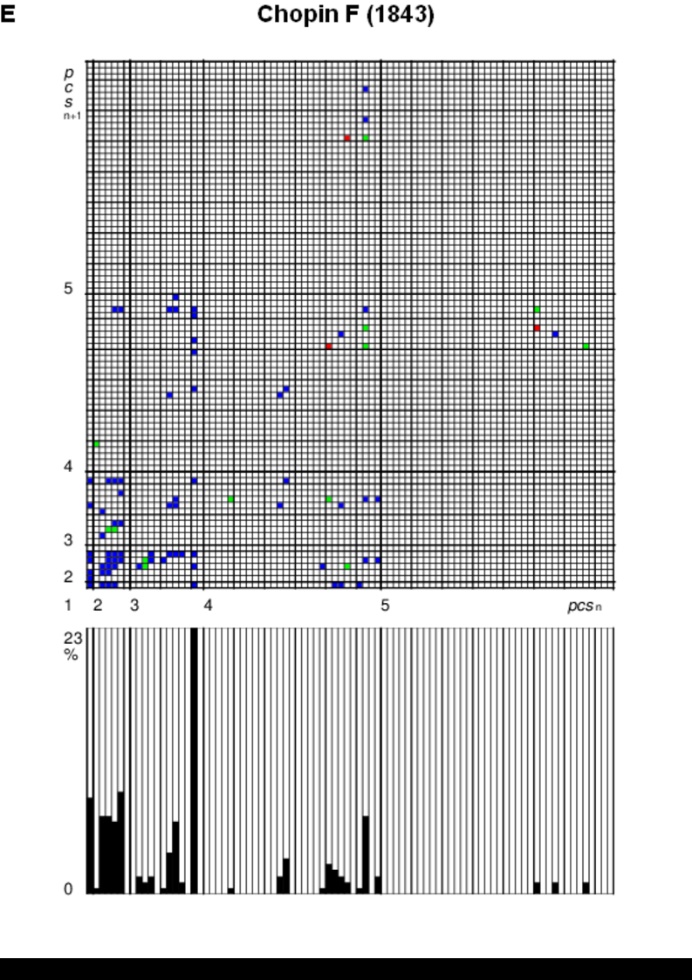

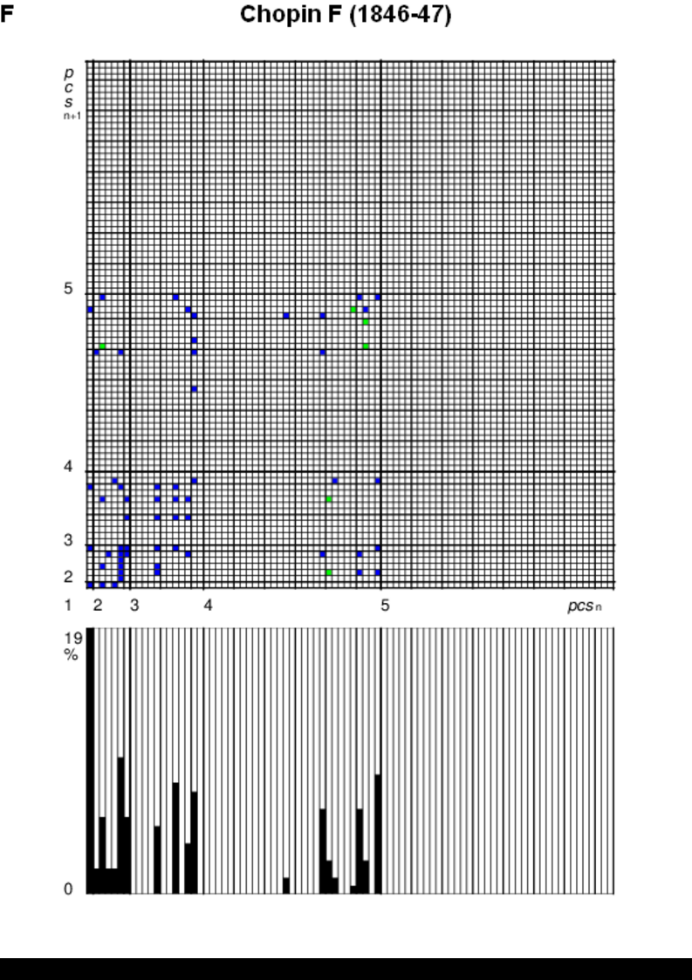

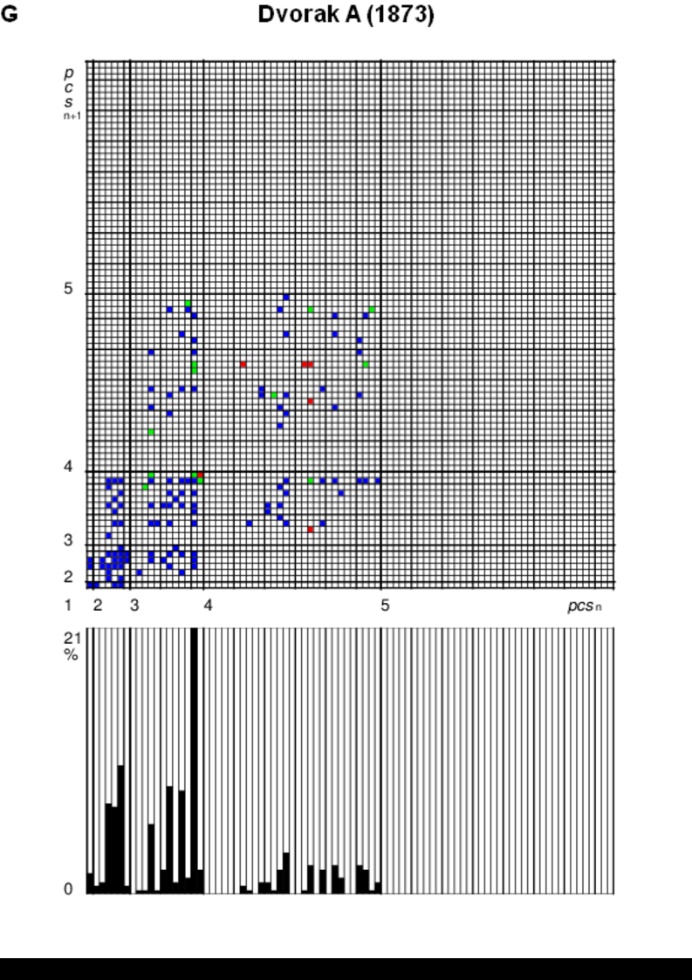

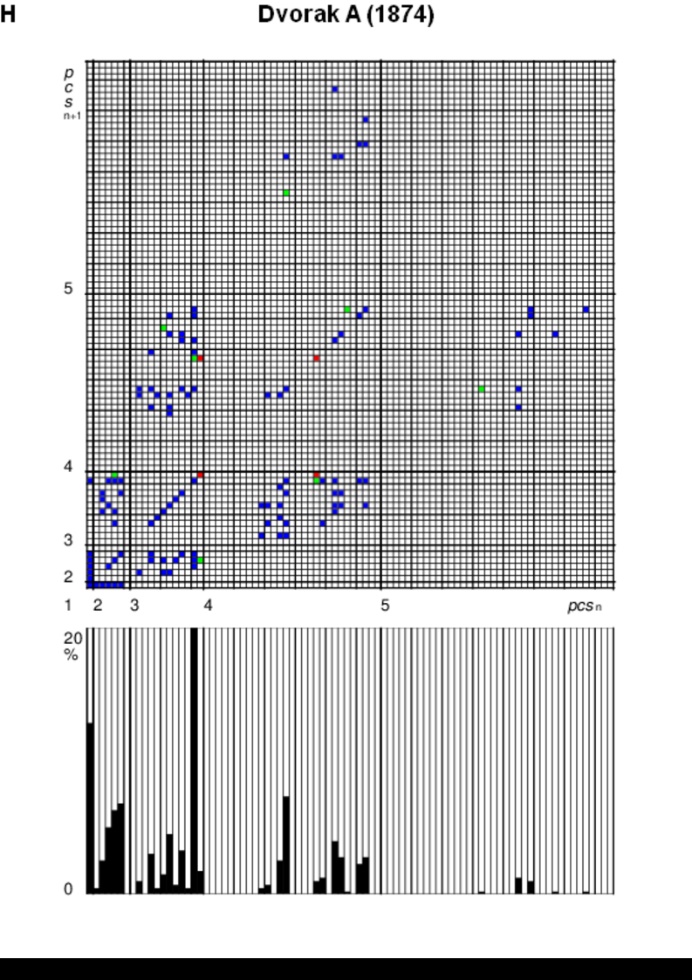

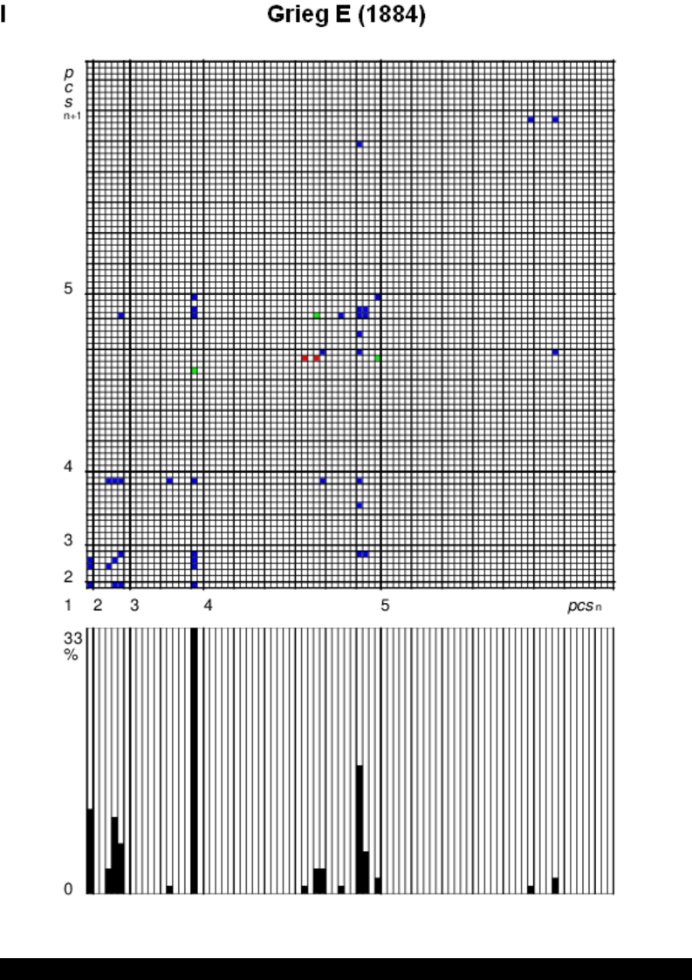

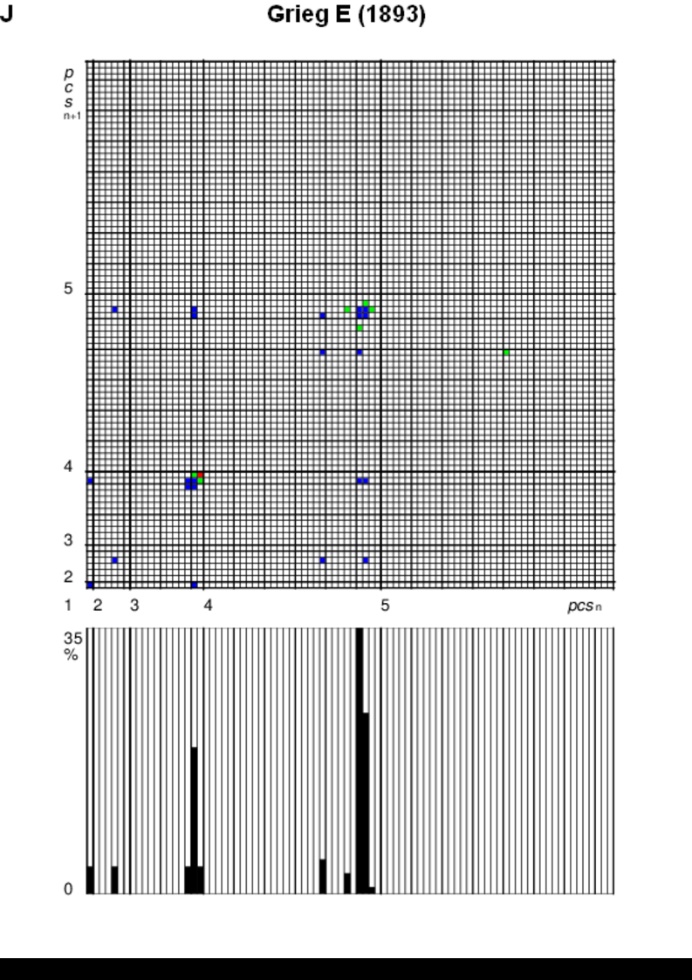

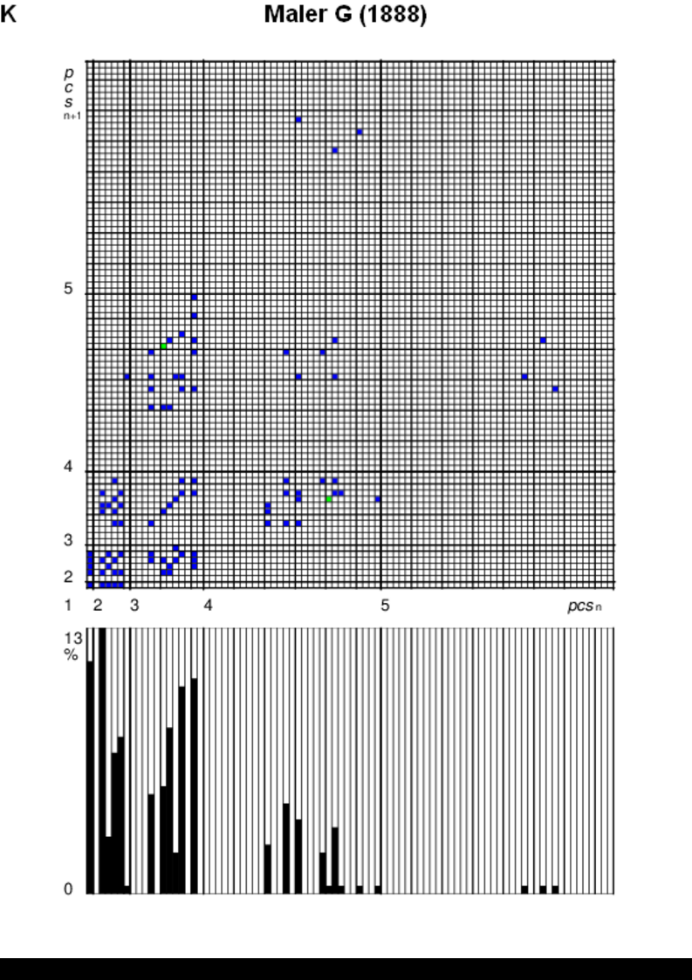

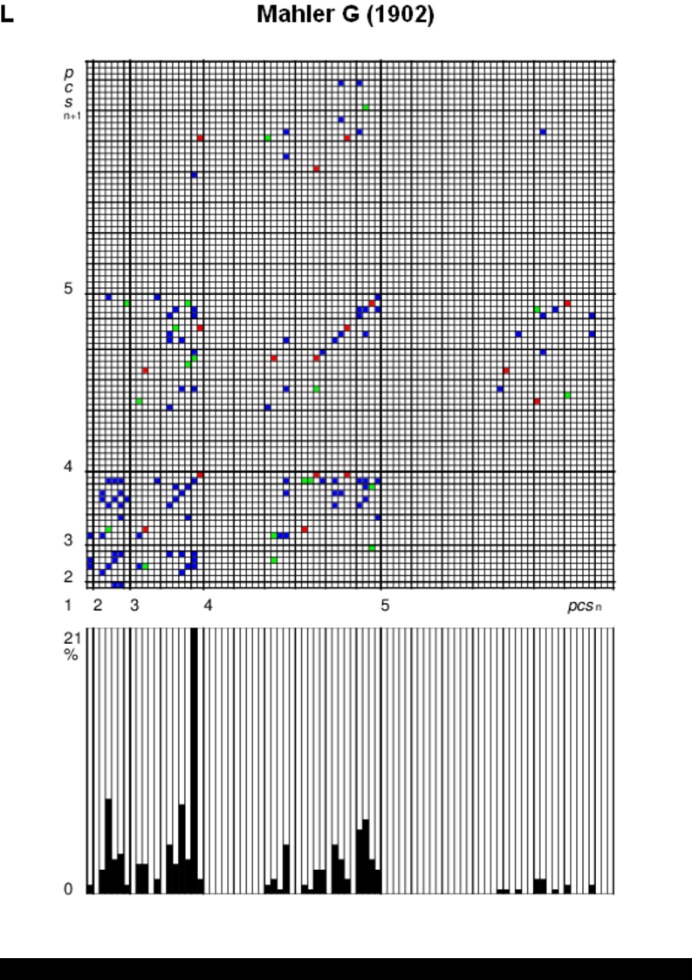

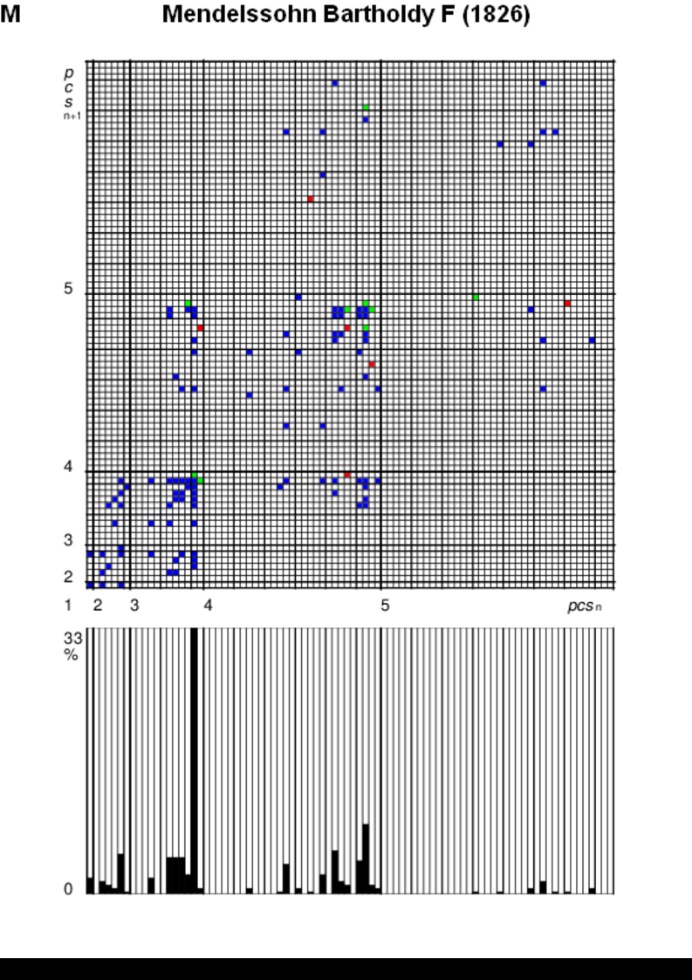

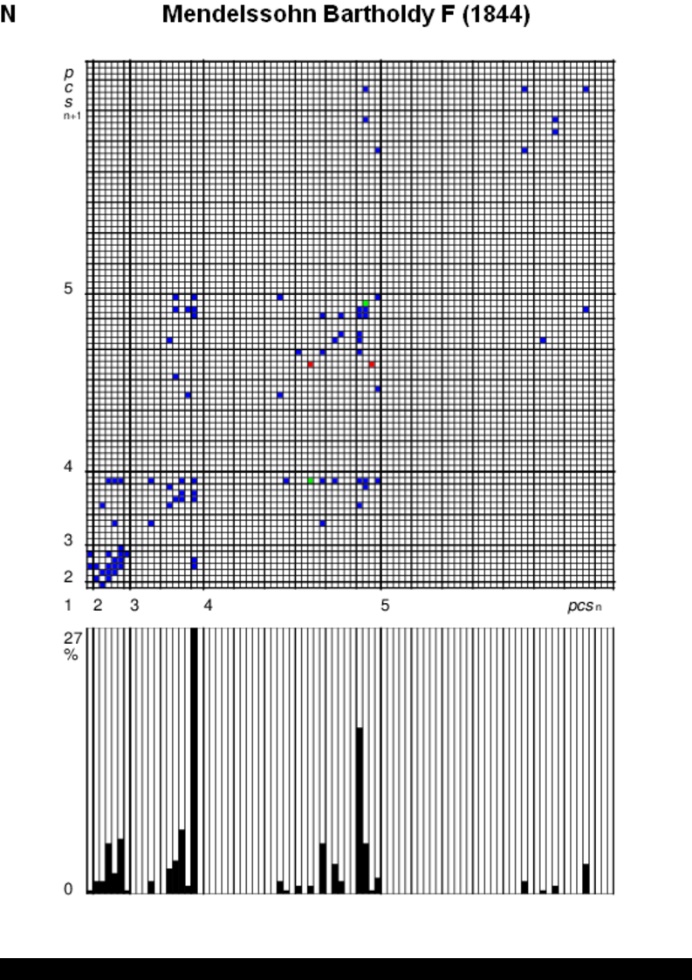

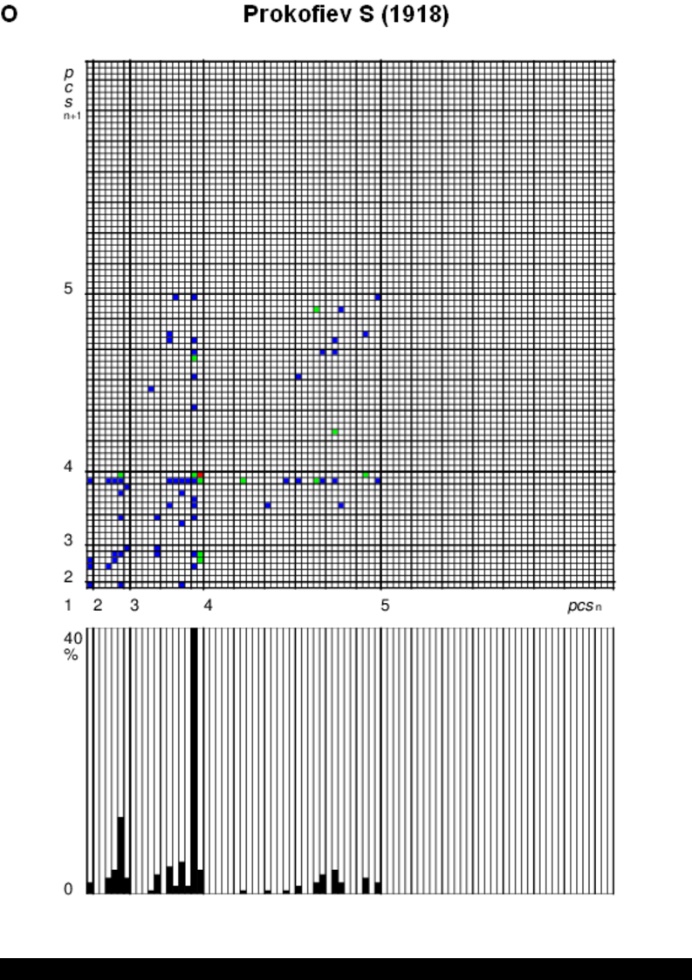

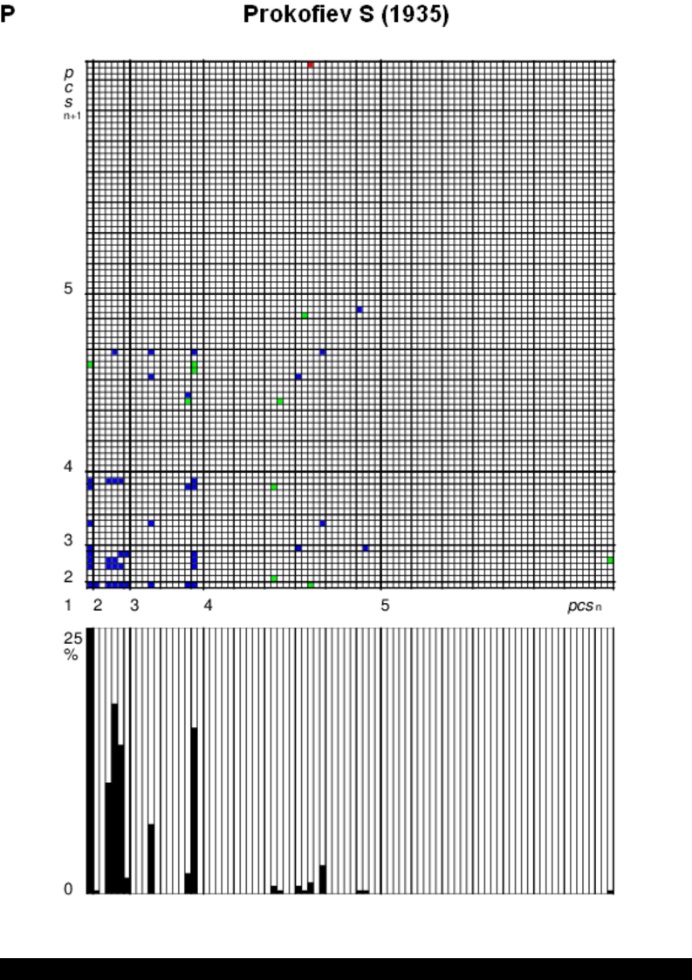

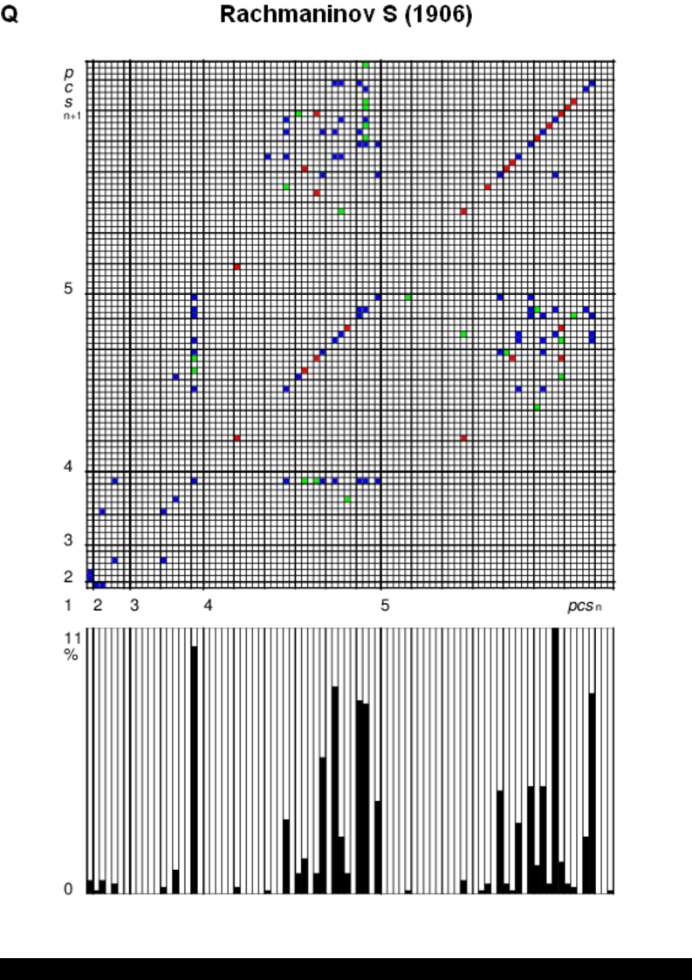

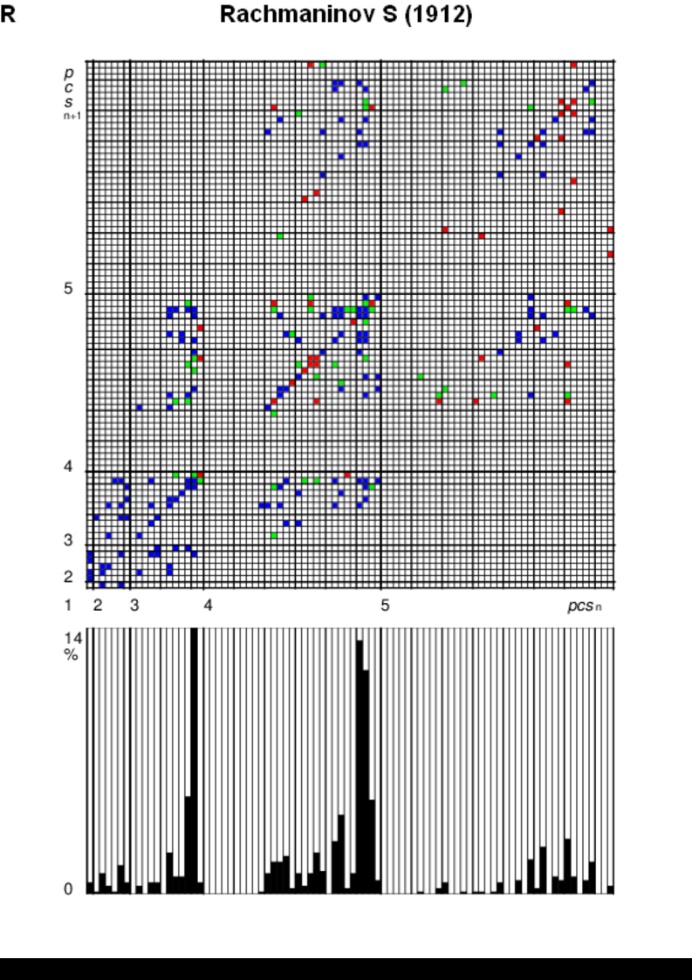

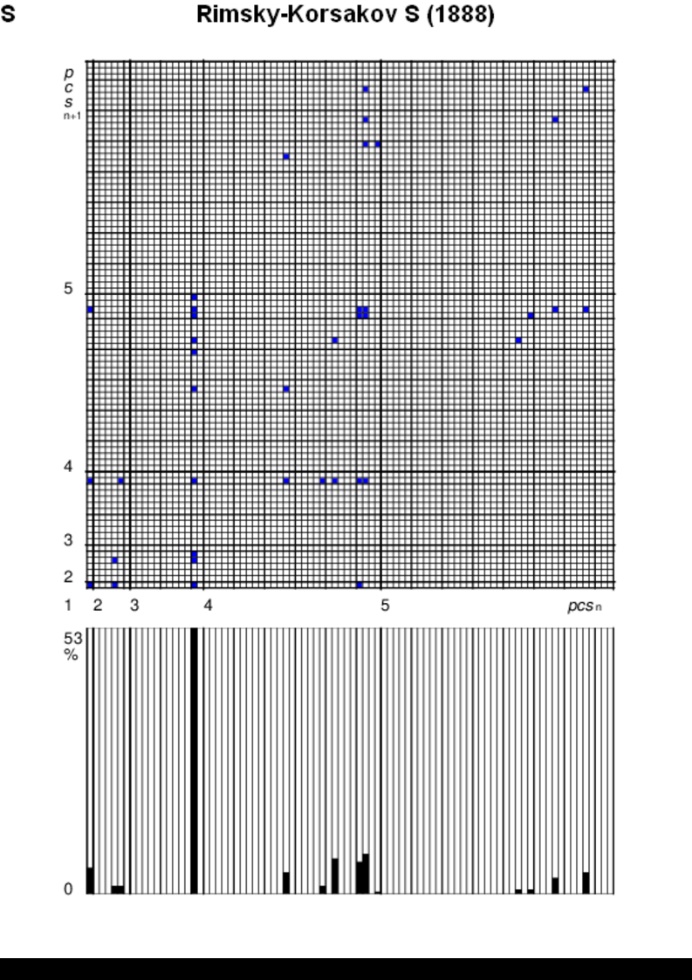

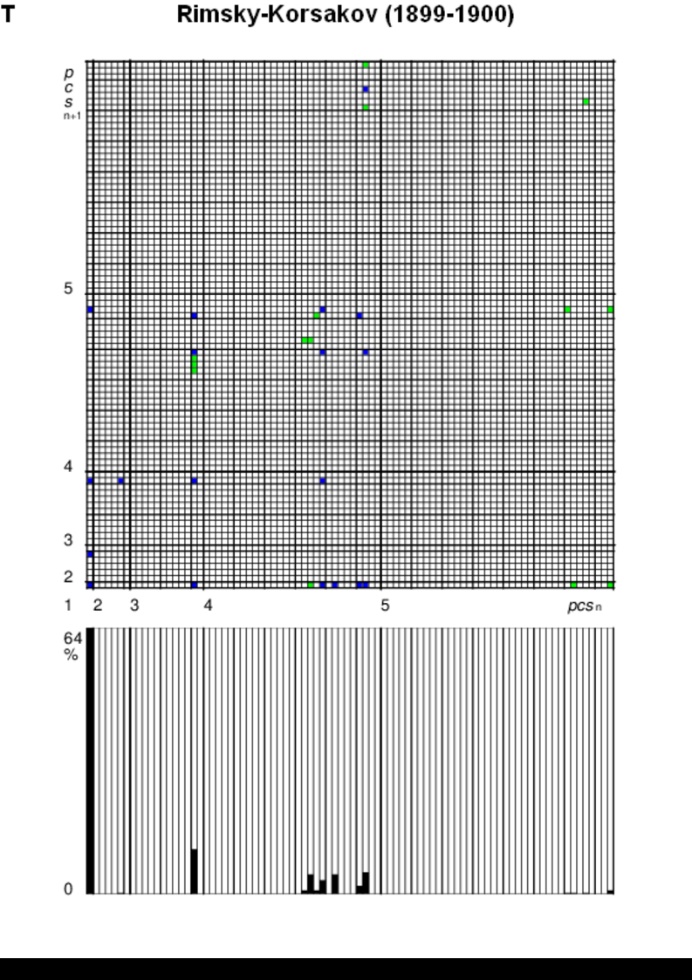

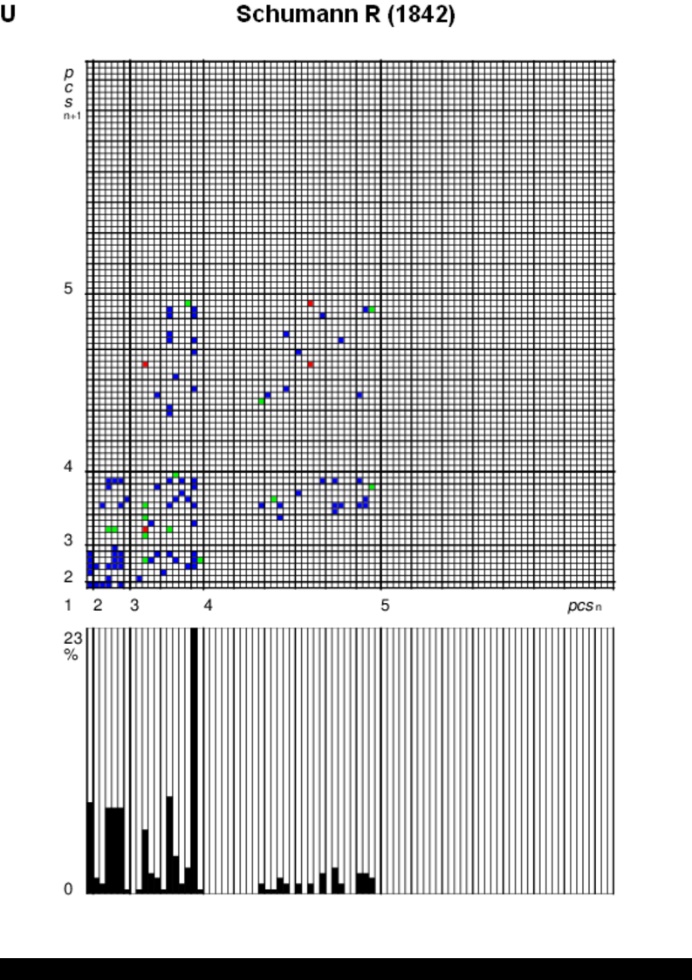

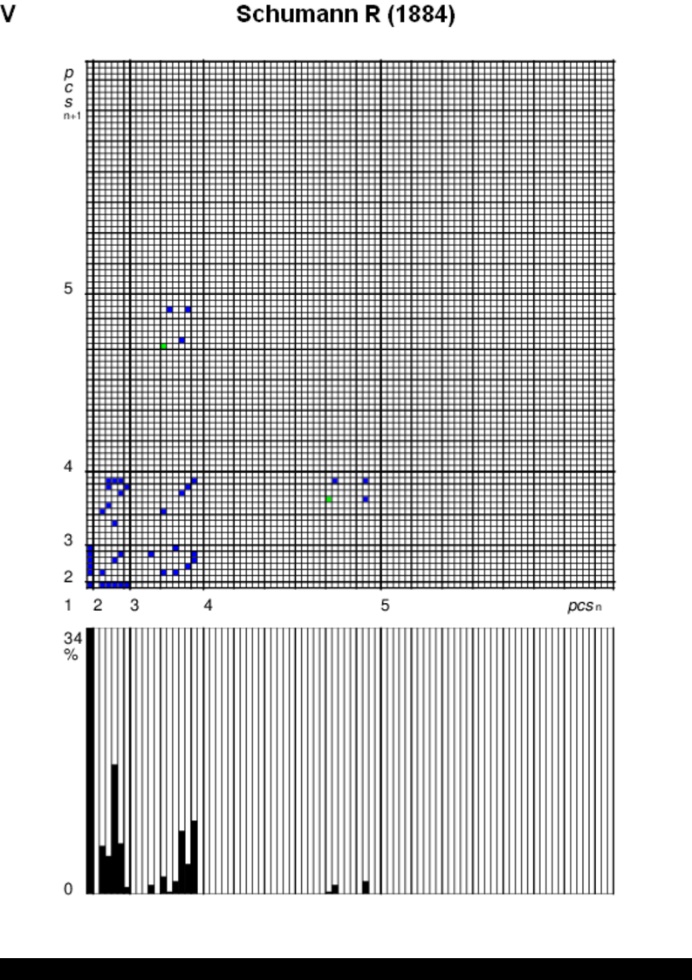

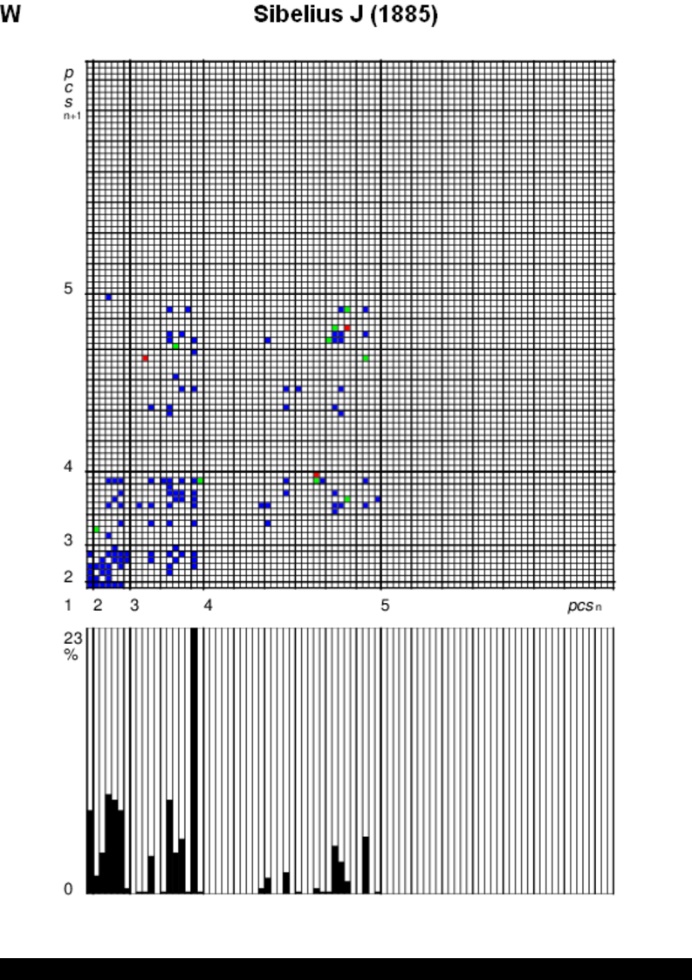

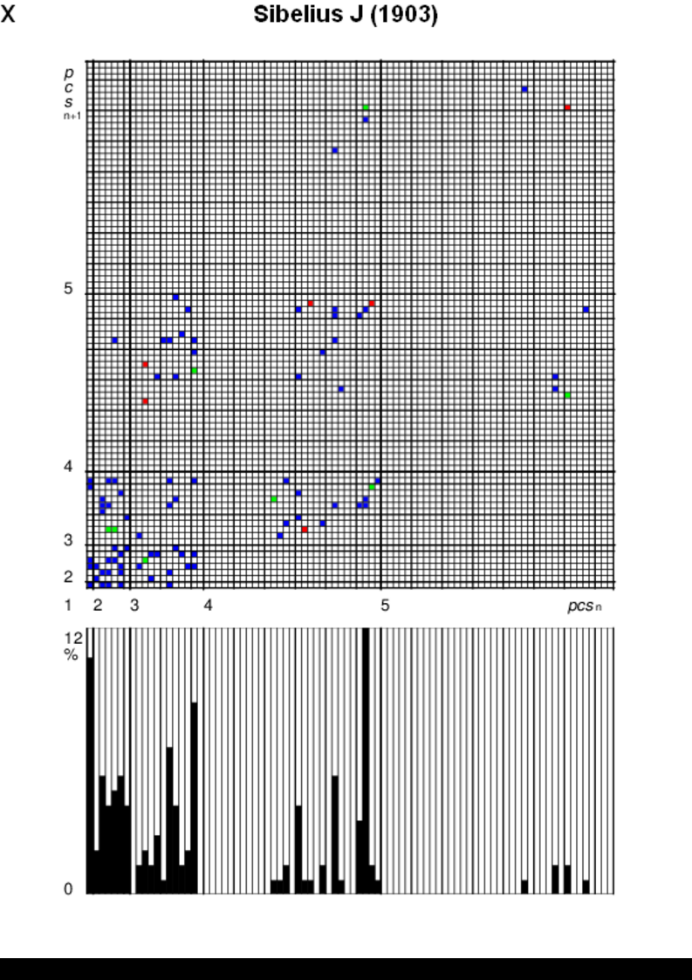

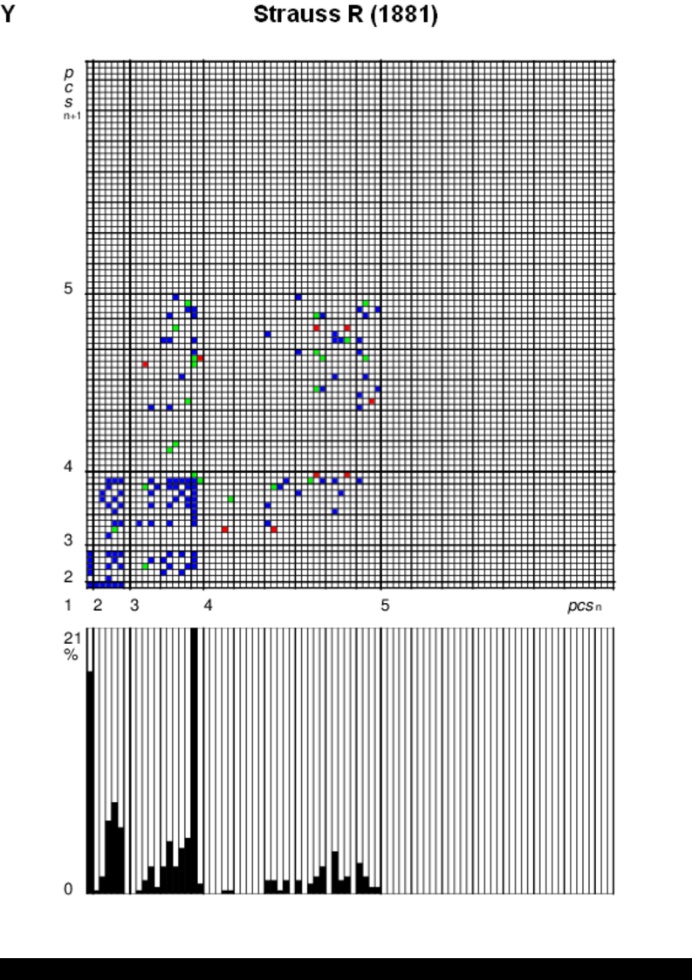

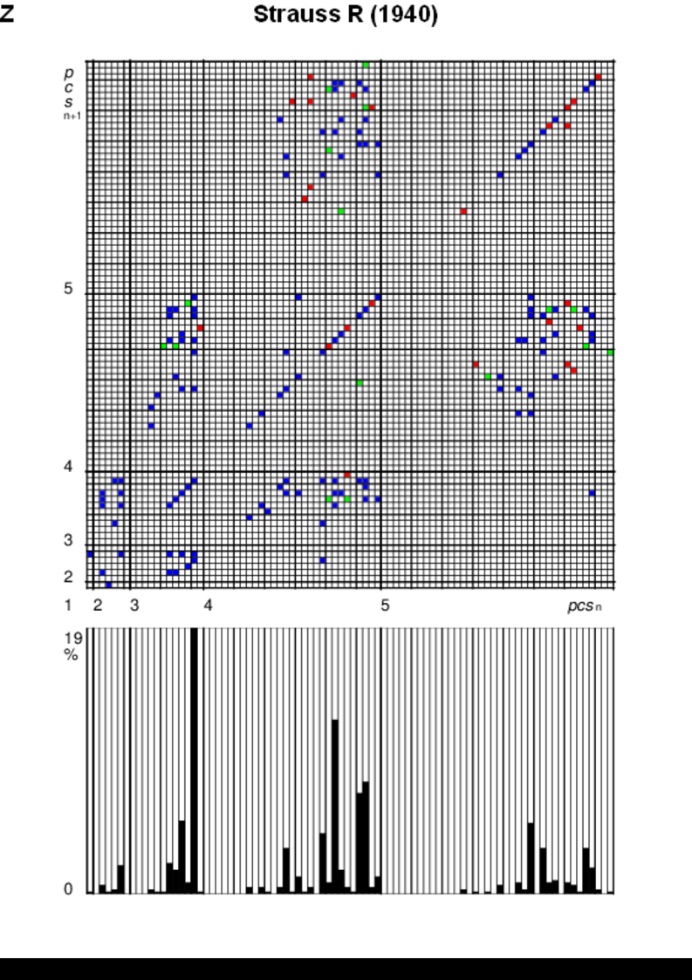

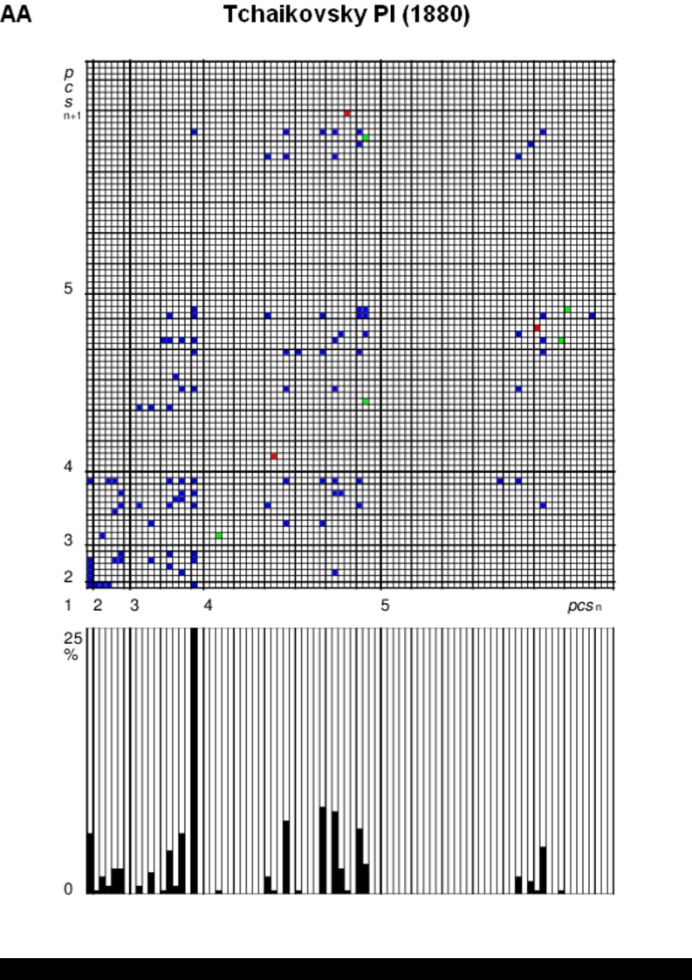

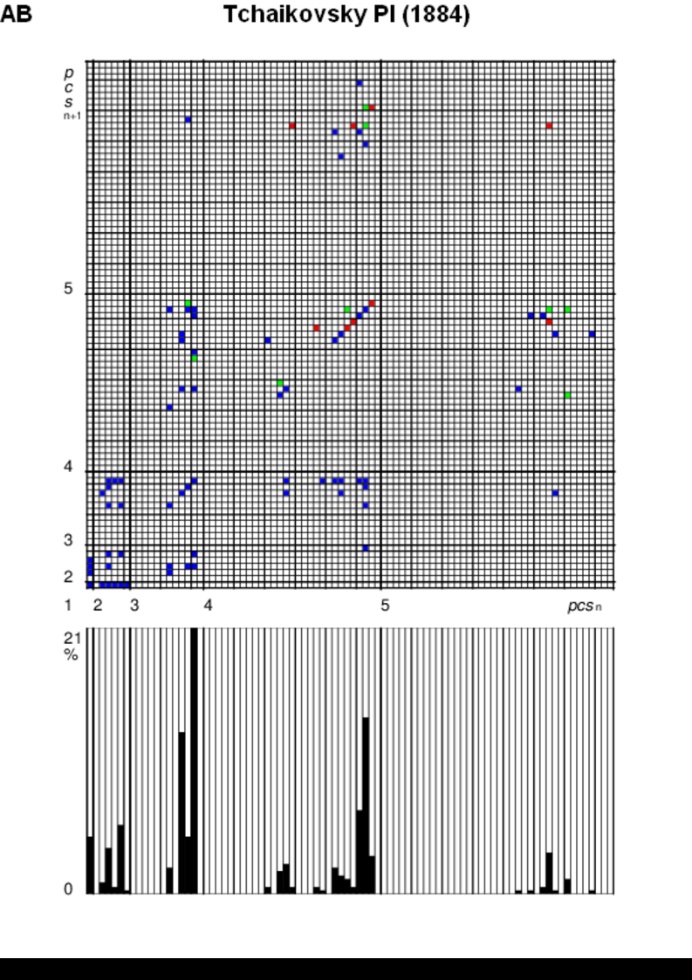

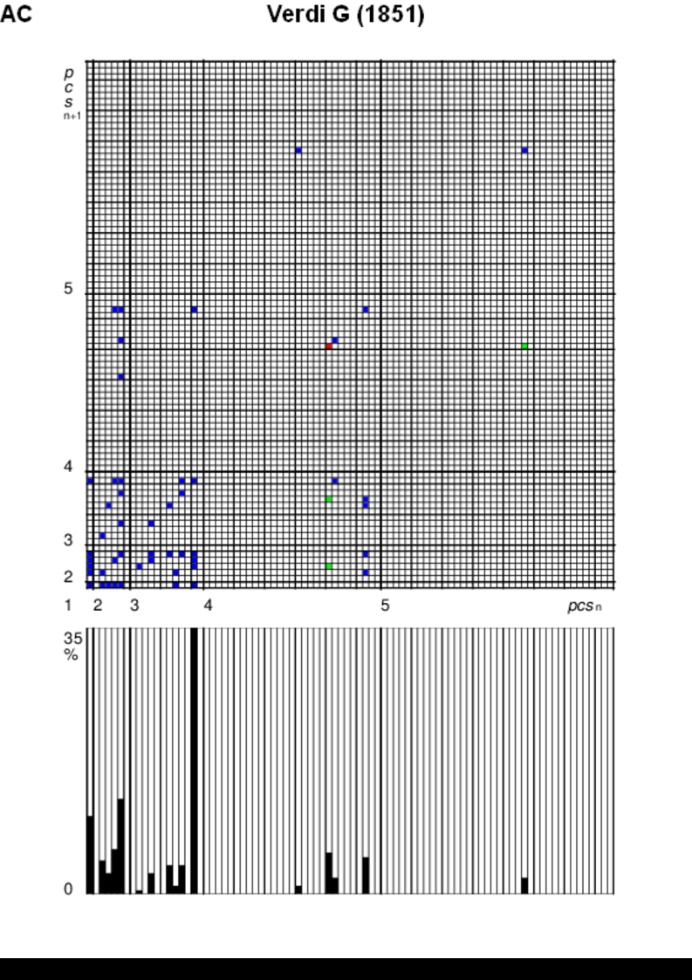

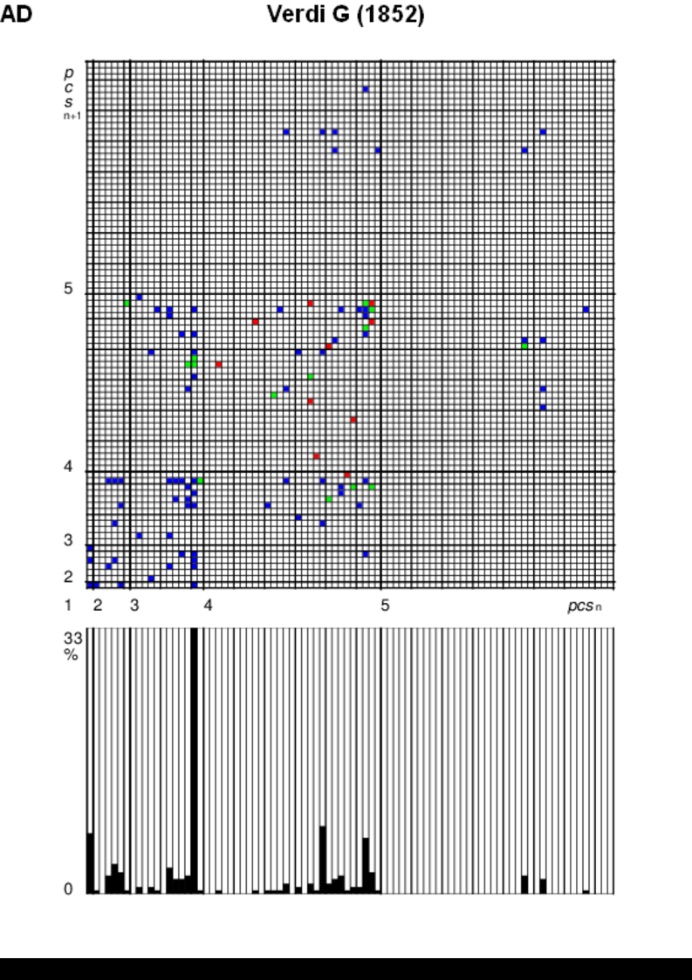

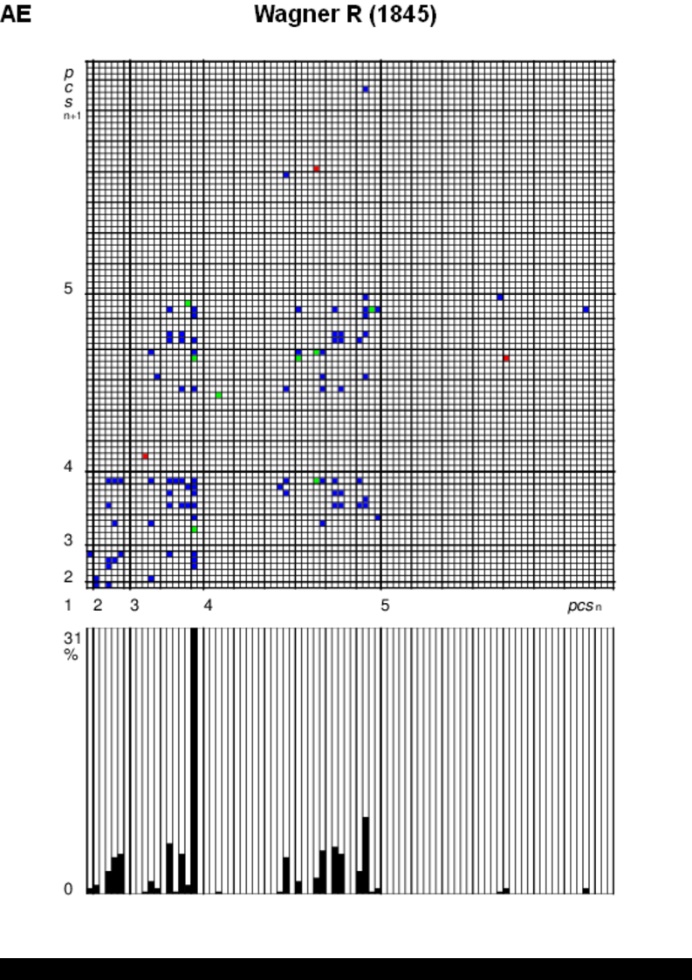

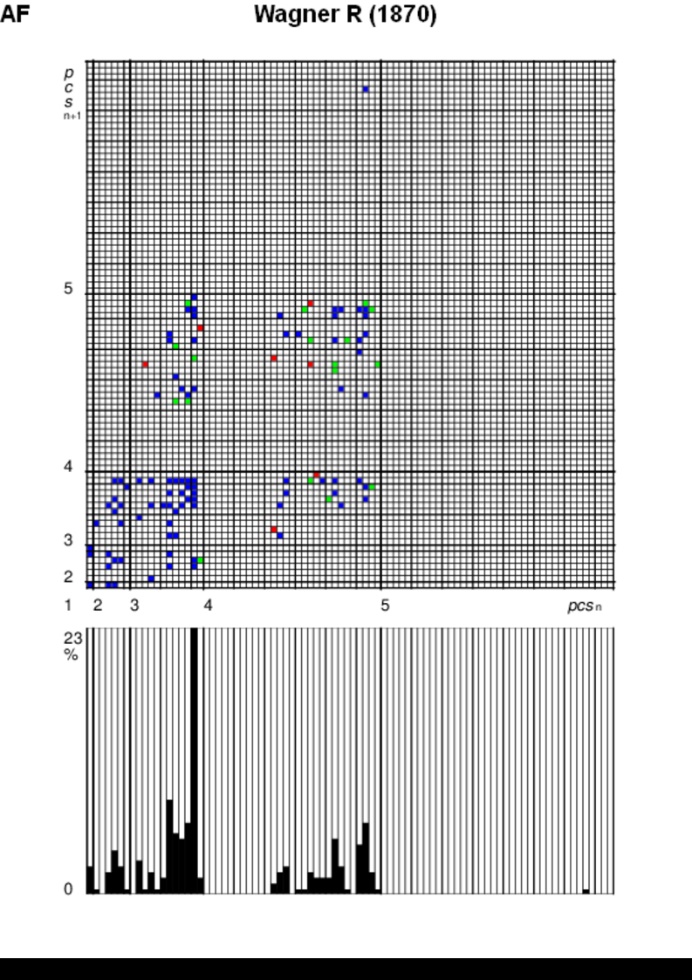
**
